# Mapping residual malaria transmission in Vietnam

**DOI:** 10.1016/j.lanwpc.2025.101545

**Published:** 2025-04-10

**Authors:** Michael A. McPhail, Yalemzewod Assefa Gelaw, Xuan Thang Nguyen, Win Han Oo, Freya J.I. Fowkes, Duc Thang Ngo, Thi Hong Phuc Nguyen, Tasmin L. Symons, Dan J. Weiss, Peter W. Gething

**Affiliations:** a The Kids Research Institute Australia, Perth, WA, Australia; b National Institute of Malariology, Parasitology and Entomology, Ministry of Health, Hanoi, Vietnam; c Burnet Institute, 85 Commercial Rd, Melbourne, VIC, Australia; d Centre for Epidemiology and Biostatistics, Melbourne School of Population and Global Health, University of Melbourne, Melbourne, VIC, Australia; e Department of Epidemiology and Preventive Medicine, Monash University, Melbourne, VIC, Australia; f Hanoi University of Public Health, Hanoi, Vietnam; g School of Population Health, Curtin University, Perth, WA, Australia

**Keywords:** Malaria, Geospatial modelling, Receptivity, Vulnerability, Risk map

## Abstract

**Background:**

Vietnam, as one of the countries in the Greater Mekong Subregion, has committed to eliminating all malaria by 2030. Declining case numbers highlight the country's progress, but challenges including imported cases and pockets of residual transmission remain. To successfully eliminate malaria and to prevent reintroduction of malaria transmission, geostatistical modelling of vulnerability (importation rate) and receptivity (quantified by the reproduction number) of malaria is critical.

**Methods:**

Case data from 2019 to 2022 was used to train a range of network and geostatistical models, from which strategically useful metrics were computed. These metrics include vulnerability, which was estimated from the abundance of imported cases, and receptivity, which was estimated based on a transmission model linking cases as well as environmental covariate data.

**Findings:**

There is considerable spatiotemporal heterogeneity in the computed metrics. Importations are concentrated in the South Central Coast and Central highlands regions. The importation rate of *Plasmodium falciparum* is around 2.5 times higher than that of *P. vivax.* The mean computed reproduction number is less than one, which is consistent with the historical decline in cases and eventual elimination. There are, however, places where the estimated reproduction number can temporarily exceed one, which explains the seasonal case resurgence observed in the case data. The reproduction number is typically higher in forested areas.

**Interpretation:**

Receptivity and vulnerability to malaria is spatiotemporally heterogeneous in Vietnam. Despite the average reproduction number being less than one, the spatial pockets and temporal windows of elevated reproduction number could prevent timely elimination of malaria or even lead to a reversal of progress. The predictive maps presented in this paper can inform appropriate intervention strategies to advance goals of malaria elimination.

**Funding:**

This work was supported, in whole or in part, by the 10.13039/100000865Bill & Melinda Gates Foundation [INV-055192 and INV-009390/OPP1197730]. The conclusions and opinions expressed in this work are those of the author(s) alone and shall not be attributed to the Foundation. Under the grant conditions of the Foundation, a 10.13039/100026877Creative Commons Attribution 4.0 License has already been assigned to the Author Accepted Manuscript version that might arise from this submission. Please note works submitted as a preprint have not undergone a peer review process. This work also includes funding support from the 10.13039/100015539Australian Government, 10.13039/501100000925National Health and Medical Research Council (Award No: GNT2025280) and Telethon Trust, Western Australia.

**Research in context**

**Evidence before this study**

We searched PubMed using the terms “malaria”, “elimination”, “geospatial”, “network”, and “model” to identify studies that have used network or geospatial models to map residual malaria in near-elimination settings; i.e., those that are data constrained by few malaria cases. We identified 35 relevant studies, but only 4 of these studies derive metrics from individual case data. One study includes an analytic framework for mapping three quantities from case data that the World Health Organisation deems important for maintaining malaria free status: receptivity, vulnerability, and malariogenic potential. Some studies infer receptivity, but not the other two quantities. There are also studies outside of this search scope that consider case-data in near-elimination settings, but use statistical methods unsuitable for producing mapped outputs. We did not identify any studies that produce such maps for Vietnam, that explain spatially varying intra-annual fluctuation in malaria risk, or account for the data bias inherent in individual case data.

**Added value of this study**

We produce the first maps of these strategically useful quantities (receptivity, vulnerability, and malariogenic potential) from reported case data for Vietnam, and outline the subsequent implications for eliminating malaria. In addition, we advance existing methodologies for inferring the key metrics from case data, which is of particular relevance to other near-elimination settings. These advances include a temporally dynamic geospatial model for receptivity that can capture seasonal variation, a more robust vulnerability model, and a novel method for adjusting for observation bias in reported case data. Our models provide additional insights into transmission chains between cases and the environmental drivers of malaria-receptivity variation in Vietnam.

**Implications of all the available evidence**

Decreasing case numbers and low estimated reproduction numbers indicate that Vietnam is trending towards eliminating of malaria. There are risks to progress including case importation and local pockets of higher receptivity. Achieving elimination will involve targeting vulnerable areas with monitoring and awareness campaigns to detect and manage imported cases. It will also involve interrupting transmission in receptive pockets with residual transmission.

## Introduction

Vietnam is aiming to eliminate malaria by 2030 and has made considerable progress towards this target. Thirty years ago, Vietnam recorded over one million cases per year.^1^ A national malaria control program was established in 1991 that included mosquito net distribution, twice yearly indoor residual insecticide spraying, intensive ad-hoc health education, and enhanced case management.^2^^,^^3^ In the six years following the introduction of the program, reported malaria deaths decreased by 97% and cases by 59%.^3^ In 2020, Vietnam reported 1420 cases of malaria, 303 of which were identified as imported.^4^ Contemporary transmission is most associated with remote locations (such as forests) and mobile labor-intensive industries (such as mines and plantations).^5^ Malaria cases since 2020 have primarily been reported in 20 provinces, each of which have recorded more than 23 cases over the past four years. Among these, the six provinces with the highest concentration of malaria cases are Gia Lai, Khánh Hòa, Lai Châu, Phú Yên, Bình Phước, and Đắk Lắk.

Progress towards elimination in Vietnam mirrors that in the wider Greater Mekong Subregion (GMS). The number of reported malaria cases in the GMS decreased by 77% and deaths by 97% between 2012 and 2022.^6^ China was certified malaria free in 2021^7^ after having no locally acquired cases since 2017. However, progress in this region is threatened by factors such as anti-malarial drug resistance, armed conflict, climate change, operational challenges due to safety and security concerns, and cross-border spill-over.^6^^,^^8^^,^^9^ Despite these threats, the GMS countries remain committed to eliminating *Plasmodium falciparum* by 2025 and all human malaria by 2030.

For malaria to be eliminated in Vietnam the remaining pockets of residual local transmission must be identified and suppressed. Additionally, the risks for the reestablishment of local transmission and progress reversal must be understood and mitigated. One such risk includes reintroduction through importation, which could result in high transmission returning to regions of high receptivity. There is a need to quantify these risks from the available data so that ministries of health or national control programs (such as the National Institute of Malariology, Parasitology and Entomology in Vietnam) can develop well informed intervention plans.

Near-elimination settings require different monitoring and intervention strategies compared to high burden settings. Within regions of generally low transmission, small foci of more intense transmission can sustain malaria and prevent elimination.^10^ Targeting parasites and vectors in transmission foci is a recommended intervention to accelerate progress towards elimination.^11^ Cases imported from endemic regions, or regions with local transmission, can be a major source of cases in places with low levels of local transmission, thereby contributing to sustained endemicity.^12^^,^^13^ Moreover, imported cases can reintroduce malaria to regions where it had previously been eliminated, with the highest risk from reintroduction found in more receptive settings.

In near-elimination settings mass intervention programs, those which are generalised across a population within a specified geographical area, may be less cost-efficacious^14^ compared to targeted control strategies underpinned by a detailed understanding of factors driving local transmission. Accordingly, the World Health Organisation (WHO) makes conditional recommendations for “targeted” and “reactive” strategies in the final phase of elimination, and conditional recommendations against “mass” strategies.^15^

Population-level metrics for monitoring malaria burden that are commonly used in endemic settings become impractical in near-elimination settings.^16^ Accurate infection prevalence measurements require increasingly large sample sizes as prevalence decreases, becoming inadequately sensitive when parasite rate drops below around 3%.^17^ Likewise, the entomological inoculation rate (EIR) is challenging to estimate in low transmission settings because finding an infected vector is “near impossible”.^16^ Serological methods can provide insight into historical exposure and have been assessed in some low transmission settings.^18^^,^^19^ However, serology as a tool for surveillance is not routinely implemented beyond research by national programs.^20^ The inapplicability of these widely used metrics in near-elimination settings motivates the development of alternate, more appropriate methods, metrics, and modelling frameworks.

In response to the unsuitability of population-level metrics, alternate frameworks have been developed using metrics more suited to near-elimination contexts. Churcher et al. (2014)^21^ presented some simple metrics that can be used to evaluate the success of different elimination strategies. These metrics require only routinely-collected data (the numbers of local and imported cases detected by surveillance systems) and are useful when local data are not collected. Reiner et al. (2015)^22^ defined and mapped receptivity, vulnerability, and malariogenic potential, which provide valuable information for the last stages of elimination.

The definition of receptivity for malaria can depend on the context.^23^ It is sometimes measured by the vectorial capacity,^24^^,^^25^ but here we use the effective reproduction number $R_{e}$ as a measure of receptivity. The effective reproduction number is the expected number of secondary infections from a single source.^22^ If $R_{e}$ is greater than one we expect the number of cases to grow exponentially and if $R_{e}$ is less than one we expect case numbers to drop and eventually reach zero. In contrast to the basic reproduction number $R_{0}$ and the controlled reproduction number $R_{c}$, $R_{e}$ is not a theoretical measure of transmission potential.^23^ Instead, $R_{e}$ is a measure of observed transmission, where the myriad factors that influence transmission (e.g., control initiatives, environment, etc.) will influence the outcome, but are unquantified. Throughout this paper ‘receptivity’ will refer specifically to $R_{e}$. It is important to note that $R_{e}$ reflects, without quantifying, the impact of the intervention strategies employed throughout the observation window. As such, $R_{e}$ is subject to change if the control initiatives are changed.

The rate at which cases are imported into a region was referred to as vulnerability by Reiner et al. (2015).^22^ The vulnerability of a setting is independent of its receptivity, and the underlying drivers of these respective risk factors can be different. The product of receptivity and vulnerability is the malariogenic potential, which measures the expected number of cases that directly follow imported cases (i.e., not factoring in subsequent local transmission). The WHO Global Technical Strategy (2021) advises that to ensure successful maintenance of malaria free status, the degree of vigilance that needs to be applied varies according to the vulnerability and receptivity of an area.^11^

In this paper we use network and geostatistical modelling techniques, trained on data provided by the Vietnamese Ministry of Health, to produce fine-scale maps (with resolution of 5 km × 5 km) of metrics that quantify the components of malaria risk in Vietnam: the effective reproduction number, vulnerability, and malariogenic potential. These quantitative, geographical decompositions of malaria risk provide actionable insights beyond what can be gleaned from raw case data. The modelling framework we use advances that of Reiner et al. (2015)^22^ and is applicable in other near-elimination settings.

## Methods

**Fig. 1:**
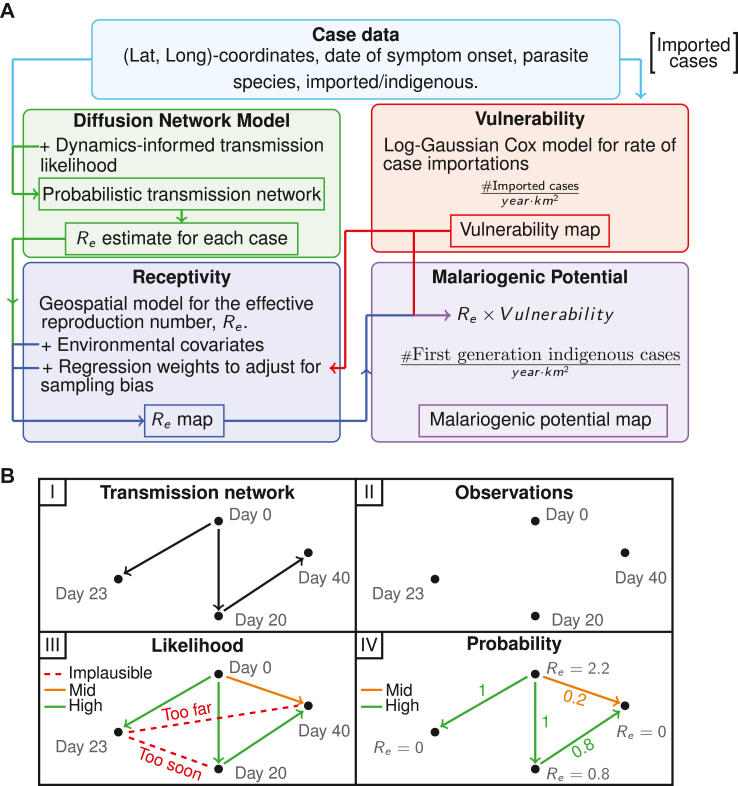
(A) A Schematic of data and prediction flow between the models used to estimate each metric. Each model is fit for each parasite separately. Only imported cases are seen by the log-Gaussian Cox model for vulnerability. (B) An illustration of the stages of inferring the diffusion network model. Beginning with (I) an unobserved transmission network, (II) the observations of subsequent cases (spatiotemporally located), (III) the imposition of transmission-likelihood relationships between the cases, and (IV) the conversion of these likelihoods to probabilities and subsequent computation of an $R_{e}$ estimate for each case.

Estimating receptivity, $R_{e}$, and vulnerability requires multiple statistical models. Each model uses different subsets of the case data and some use outputs from other models as inputs. The schematic in Fig. 1 gives a high-level overview of how information flows from case data to malariogenic potential predictions. The diffusion network model and the vulnerability model can be inferred from the case data alone, and the output of both of these models informs the geospatial model for $R_{e}$. The vulnerability and $R_{e}$ predictions are then combined to predict the malariogenic potential. In this section we provide more details about the input data and the respective models. More complete descriptions of each of the models listed here, as well as model validation and sensitivity analysis can be found in Supplementary Materials B–F.

### Data

**Fig. 2:**
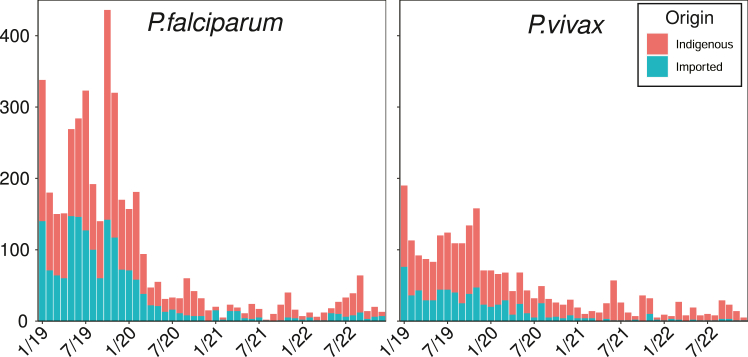
Stacked bar charts illustrating the total number of reported cases of *P. falciparum* and *P. vivax*, separated by importation status.

Between 2019 and 2022, Vietnam recorded 7009 malaria cases. Of these 6704 had complete reported information, including 4247 caused by *P. falciparum* and 2380 caused by *P. vivax*, which are the two species we consider in this paper. A full breakdown of the available data is given in Table 1. While the results presented in this paper are based only on data from the years 2019–2022, the malaria situation in Vietnam has not changed drastically since this time period and Vietnam is still near elimination. The Ministry of Health designate each recorded case as either indigenous or imported based on the travel history of the infected individual prior to the onset of symptoms. Imported cases were suspected infections originating from outside of the region where the case was reported, either nationally (i.e., the case originated from outside the Commune of reporting) or internationally (the case originated outside of Vietnam). There were 1676 imported *P. falciparum* and 702 imported *P. vivax* cases during the study period, of which 5% were imported internationally. Only a small number of cases in the data provided by the Ministry of Health were labelled as recurring or relapsing infections based on the time since the patients last infection. Less than 0.2% of *P. vivax* infections were labelled as relapses, which suggests that some portion of relapsed *P. vivax* cases have been labelled as new infections. The implications of misclassified relapses will be covered in the discussion. We assume infections are either new or relapse, and do not consider multiclonal infections. Each case was geolocated and the date of symptom onset was recorded. For geospatial modelling our target data are points specified by latitude and longitude. The monthly number of reported *P. falciparum* and *P. vivax* cases, distinguished by importation status, are illustrated in Fig. 2A.

**Table 1:** Nationally reported cases by designation.

Designation	Total	*Falciparum*	*Vivax*	Malarie	Ovale	Mixed
All	6704	4247	2380	30	6	41
Indigenous	4296	2571	1678	18	0	29
Imported	2408	1676	702	12	6	12
Internal import	2186	1530	638	5	1	12
GMS	112	57	54	1	0	0
International	101	84	6	6	5	0
Recurrence/Relapse	9	5	4	0	0	0

### Diffusion network

The description of the transmission dynamics here follows that of Routledge et al. (2020),^26^ based on the diffusion network model described in Gomez-Rodriguez et al. (2011),^27^ with the extension to a spatial kernel described in Routledge et al. (2021).^28^ A summary of the model is described below, and a more complete technical description can be found in Supplemental Information Section B. Also note that we fit a diffusion network model for each *Plasmodium* parasite separately.

The schematic in Fig. 2B illustrates the key stages of the network model using a simple transmission chain as an example. The cases are linked by transmission; however, the observations only include dates and locations of cases, not the edges linking cases. We assume that any case can be a source, but only cases designated indigenous can be infected by sources in the data set. A transmission likelihood model (detailed below) is used to calculate the likelihoods of each pair of cases being related by transmission. These likelihoods form the edge-weights of a directed network where an edge indicates a potential infection event. The incoming edges for each case are normalised to sum to one, transforming the edge-weights into probabilities for each source node infecting each target. The sum over the outgoing edges for each case gives the expected number of infections from each source; that is, the effective reproduction number for that case, $R_{e}$.

The likelihood of a transmission event linking two cases is assumed to depend only on the time and distance separating them. We assume that each observed malaria case is the result of infection from a single source, and that a case cannot be the source of infection for any other case whose symptoms begin less than 15 days after symptom onset for the original case. cases cannot be directly linked if symptom onset occurs within 15 days.^26^ The time between the onset of symptoms for the source case and the infected case is known as the serial interval, which is a combination of the following random processes: (a) the time between symptom onset and infectiousness, (b) the period of infectiousness, (c) the time between infectiousness onset and human to mosquito transmission, (d) the incubation period of the parasite in the mosquito, (e) the time taken for a mosquito to infect a human, and (f) the period of infectiousness for a mosquito.

For potential sources of infection, we assume the time-dependence of the likelihood of infection is given by a shifted Rayleigh distribution.^26^ The likelihood of transmission is set to zero for cases less than 15 days before an infection and is given by the Rayleigh distribution for cases 15 days or more before an infection. This distribution encapsulates the myriad processes influencing the time between observed infections. Our prior assumption on the shape of the Rayleigh distribution includes an expected serial interval of 33 days.

We assume that the likelihood of infection decreases with distance from a source, and this rate of decrease is the same everywhere. We use a Gaussian distribution to model this rate of spatial decay. We assume that this Gaussian distribution is centred at zero, and our prior assumption is that the length scale of decay (i.e., the standard deviation of the Gaussian distribution) is approximately 31 km. This parameterisation corresponds to the “moderate human movement” scenario considered by Routledge et al. (2021).^28^

Following the approach of Routledge et al. (2020),^26^ we also incorporate the possibility of infection from an unobserved source. Ideally, these unobserved sources are only invoked if no other observed source is plausible. The probability of infection from an unobserved source is parameterised by ϵ, which is small and assumed to be drawn from a truncated normal distribution with mean and standard deviation 0.001, and bounds of zero and one.

The edge-weight between a source and target pair of cases depends on (a) the likelihood of pairwise source—target infection given the spatiotemporal proximity of the cases, (b) the likelihood of that target not being infected by other sources until it is infected by the source, and (c) the instantaneous infection rate at the time of infection between the source and target (known as the hazard function *H*) given the separation of the cases. For (b) the survival function *S*, which is the probability of the target surviving the interaction with a source until the time of infection, is calculated for all potential sources of a target infection. Thus, the likelihood that a given source infects a given target accounts for the hazard posed by that source as well as the survival probability against all other sources of infection. These likelihoods are transformed into probabilities by normalising the incoming edges to each target node. The hazard posed by unobserved sources is small and constant, ϵ.

From the inferred diffusion network we can not only estimate $R_{e}$ for each source, but also gain insight into the possible chains of transmission that gave rise to the observed cases. This includes the identification of nodes that have no plausible source of infection within the observed cases. Each case falls into one of three categories based on it's relation to other cases: imported, indigenous (resulting from local transmission), and non-imported but with no obvious local source.

### Mapping vulnerability

We use a log-Gaussian Cox model to estimate the rate of importation of cases for each parasite. The output is an intensity surface, λ(x), where x is the location and λ has dimensions of cases per year per $km^{2}$. Integrating this surface over a region gives the expected number of importations into that region per year. For a more detailed description of the technical approach, see Supplemental Information Section C.

### Mapping receptivity: geospatial model

The predicted $R_{e}$ values for each case from the diffusion network model are used as target data in a geospatial model to estimate $R_{e}$ throughout Vietnam. For a full description of the geospatial model, see Supplemental Information Sections D and E. Here we present only the key components.

We spatially and temporally match to each observed case a suite of environmental and static covariates that are thought to influence transmission intensity. Each covariate has a resolution of 5 km × 5 km, which is the resolution on which we make predictions. We include the value of each time-dependent covariate for the month of symptom onset as well as one month before and after, which we denote by appending “lag” or “lead” respectively to the covariate name. We use a rudimentary wrapper method to reduce the full set of covariates (with leading and lagged counterparts) to a smaller, more informative set. This method sequentially drops the covariate that gives the largest overall decrease to the Akaike information criterion (AIC) until no further improvement can be made by dropping individual covariates. This is a *greedy* optimisation routine, and does not guarantee a globally optimum set of covariates.

The $R_{e}$ predictions from the diffusion network model can be partitioned into two groups: cases that are potential sources of infection ($R_{e}>0$) and those that aren't ($R_{e}=0$). To reflect this dichotomy, we use a zero-inflated geospatial model comprising two parts: a Bernoulli distribution for the probability that $R_{e}=0$ and a gamma distribution for $R_{e}>0$. The same set of environmental covariates is used for both components of the model. To fit this model we use the R package glmmTMB.

### Mapping receptivity: data bias correction

Data limitations can become increasingly prominent as elimination is approached and less cases are observed. The only available independent observations of receptivity occur where cases are imported, and there are many parts of Vietnam for which there was no imported cases. Of the places where imported cases were recorded there are two additional sources of data bias, neither of which are a consequence of a lack of data. The first source is the spatially heterogeneous rate of case importation. The seeding of cases due to organic importation of index cases will lead to some places with very few observations and others preferentially observed. The second of these sources of observation bias is the production of more cases in more receptive areas. Each case imported into a region of higher receptivity (or during a transmission-suitable window) can infect more people, generating more observations. In this case, unlike importation-driven bias, the observation bias is directly related to the process being observed. Birello et al. (2023)^29^ discuss a related issue when estimating spatially heterogeneous reproduction ratio from population-level incidence data. Not accounting for the spatiotemporal heterogeneity of observation abundance will result in biased parameter estimates.

We adjust for this observation bias using a weighted linear regression. The regression weights assigned to each observation depended on ancestry. If a case was either imported or had no known source, we determine the weight of that observation using the inverse rate of importation (updated to include orphan cases emerging from the transmission network). For the remaining cases we recursively calculate the weight of each case by combining the regression weight, the edge-weight, and the reproductive number of each potential source for that case. The last two of these quantities are provided by the diffusion network model. After computing the regression-weights of all cases, we truncated these values at the 5% and 95% quantiles to reduce the variance of our predictions. A more detailed description of the computation and testing of this method can be found in Supplemental Information Section D. The most important aspect of these regression weights is that observations in high importation areas are down-weighted relative to those in under sampled areas, and cases from highly infectious sources are down-weighted to account for the overabundance of such observations.

### Ethics approval

Ethical approval for this study was obtained from the Curtin University Human Research Ethics Office (approval number: HRE2021-0735). Ethical approval for the data was given by the National Institute of Malariology, Parasitology, and Entomology (NIMPE) Ethical Review Board (decision number 1050/QD-VSR). Permission to access the data was granted by the NIMPE in Vietnam. The NIMPE in Vietnam provided consent for this publication. Additionally, as routine surveillance data was used, individual consent for publication was not required.

### Role of funding source

The funders had no role in study design, data collection, data analysis, interpretation, or writing of this report.

## Results

The number of reported monthly *P. falciparum* and *P. vivax* cases nationwide dropped notably during the 4-year observation window (see Fig. 1). This trend is observed for both indigenous and imported cases. The definition of “imported” includes cases with a history of intra-national travel, so these values may be correlated (i.e., if there is less malaria overall, there will be less intra-nationally imported cases). There is apparent seasonality in the number of *P. falciparum* cases, with the largest numbers typically occurring in the latter half of each year. These spikes are mostly comprised of indigenous cases, which suggests temporal variations in receptivity.

**Fig. 3:**
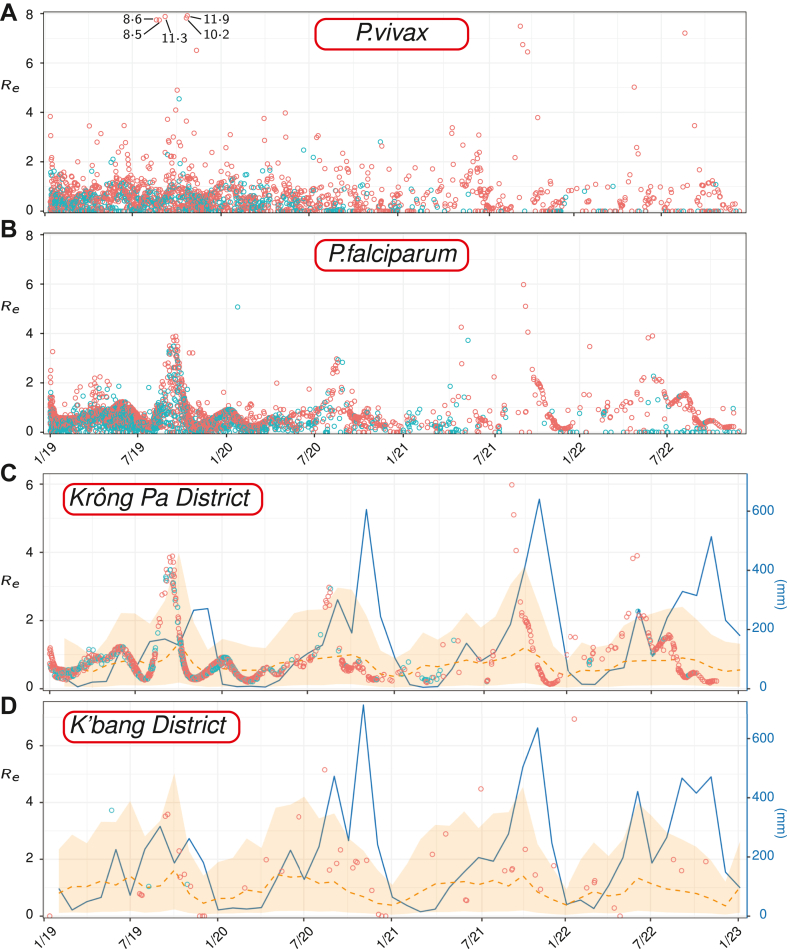
(A, B) Time series of predicted $R_{e}$ values for (A) *P. vivax* and (B) *P. falciparum* associated with all reported cases (each case is represented by a single point). Blue points are imported cases and red are indigenous. (C, D) Time-series for predicted $R_{e}$ of *P.falciparum* cases in the (C) Krông Pa and (D) K'bang districts. Rainfall (CHIRPS) is illustrated by the blue line. Circles represent predictions from the diffusion network model for imported (blue) and indigenous (red) cases. The orange dashed line represents predictions from the geospatial model and the shaded region indicates the 95% confidence interval.

The estimated $R_{e}$ values from the diffusion network model are illustrated in a time series by Fig. 3A and B. We note that there is no sustained drop in $R_{e}$ over the full observation window. Instead, the volatility in the predicted $R_{e}$ increases, and there are some higher $R_{e}$ values in 2022 than earlier years. In conjunction with the fact that there are fewer observations in later years, this suggests that lower receptivity locations are contributing fewer cases over time, and the remaining cases are more likely to be found more receptive areas.

On sub-annual timescales, there is substantial variation in $R_{e}$. For *P. falciparum*, in particular, there are apparent seasonal peaks. This result is supported by the earlier observations of seasonal peaks in indigenous cases within the raw data (Fig. 1). In Fig. 3C and D we illustrate the predicted $R_{e}$ values for each case in the Krông Pa and K'Bang districts respectively, along with the monthly total rainfall values extracted from CHIRPS^30^ rainfall estimates and population-weighted^31^ mean of the predictions from the geospatial model for $R_{e}$. There is a strong correlation between the timing of the predicted receptivity and monthly rainfall, which is indicative of a strong environmental contribution to the seasonal receptivity signal.

**Fig. 4:**
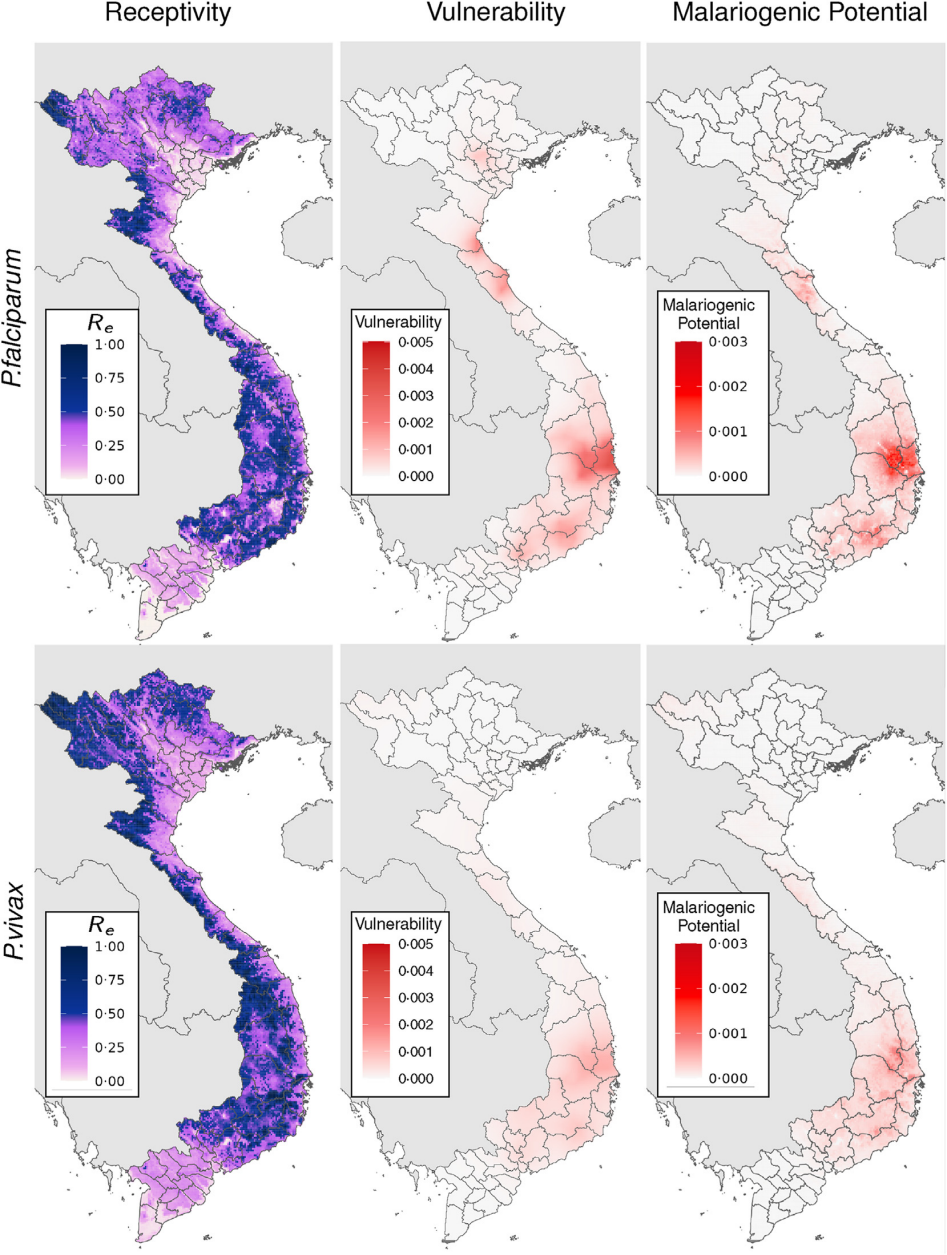
The time-averaged $R_{e}$ prediction (left column), vulnerability (middle column), and malariogenic potential (right column) during the 4-year study period for each parasite (top row is *P. falciparum* and bottom row is *P. vivax*). The units of vulnerability are number of imported cases per year per $km^{2}$, and the units of malariogenic potential are the number of first generation indigenous cases per year per $km^{2}$.

The estimated vulnerability maps for both parasite species are illustrated in Fig. 4. Vulnerability is spatially heterogeneous, with most cases being imported in the South Central Coast and Central Highlands regions. The level of vulnerability for each parasite is different with the importation rate of *P. falciparum* approximately 2.5 times higher than that of *P. vivax*.

We illustrate the time-average of the receptivity maps between 2019 and 2022 for both parasites in Fig. 4 and the fitted model parameters in Table 2. Overall, the predicted $R_{e}$ values for *P. vivax* are slightly larger than that of *P. falciparum*, but in both cases $R_{e}$ is less than one throughout the country. The regions with the lowest predicted receptivity are the Mekong River Delta the Red River Delta regions. The predicted receptivities illustrated in Fig. 4 are generally consistent with the macroscale trend of declining malaria in Vietnam. The model parameters in Table 2 indicate which factors contribute to the $R_{e}$ spatial patterns in Fig. 4. For both parasites the presence of forest cover is associated with higher $R_{e}$. The apparent seasonality in *P. falciparum* is influenced by statistically significant dependencies on rainfall, the Enhanced Vegetation Index, and the Temperature Suitability Index of *P. falciparum* in the month after the date of symptom onset (among other factors). This is consistent with the fact that the environmental conditions during the infectious period of a case influences how many subsequent infections may arise.

**Table 2:** Coefficient values and 95% confidence intervals for *P. falciparum* and *P. vivax* for the zero-inflated model.

	*P*. *falciparum (continuous)*	*P*. *falciparum (zero-inflated)*	*P*. *vivax (continuous)*	*P*. *vivax (zero-inflated)*
Intercept	−0.42 ± 0.02	−0.35 ± 0.17	−0.26 ± 0.03	−1.56 ± 0.09
Log (Precipitation) (lead)^30^	0.15 ± 0.02	0.5 ± 0.15	0.07 ± 0.03	0.44 ± 0.09
Log(Precipitation)^30^	−0.16 ± 0.03	0.3 ± 0.18	–	–
Log (Precipitation) (lag)^30^	0.06 ± 0.02	0.19 ± 0.14	0.1 ± 0.03	−0.03 ± 0.08
Vegetation index (lead)^32^	0.29 ± 0.05	−0.33 ± 0.28	0.26 ± 0.07	−0.47 ± 0.16
Vegetation index^32^	−0.16 ± 0.06	−1.25 ± 0.3	−0.23 ± 0.07	−0.04 ± 0.17
Land surface temperature (day, lead)^33^^,^^34^	–	–		
Land surface temperature (day)^33^^,^^34^	–	–	0.47 ± 0.09	−0.45 ± 0.2
Land surface temperature (day, lag)^33^^,^^34^	−0.08 ± 0.04	−1.59 ± 0.24	−0.32 ± 0.08	−0.46 ± 0.17
Land surface temperature (night)^33^^,^^34^	−0.2 ± 0.04	1.84 ± 0.22	–	–
Land surface temperature (night, lag)^33^^,^^34^	0.1 ± 0.04	−0.24 ± 0.22	–	–
Land surface temperature (diurnal difference, lead)^33^^,^^34^	0.16 ± 0.03	0.05 ± 0.18	0.14 ± 0.05	−0.78 ± 0.13
Land surface temperature (diurnal difference)^33^^,^^34^	–	–	−0.17 ± 0.07	0.38 ± 0.16
Land surface temperature (diurnal difference, lag)^33^^,^^34^	–	–	−0.05 ± 0.07	−0.48 ± 0.14
Tasselled cap brightness (lead)^32^	−0.25 ± 0.04	−0.4 ± 0.24	–	–
Tasselled cap brightness^32^	0.25 ± 0.07	1.84 ± 0.42	–	–
Tasselled cap brightness (lag)^32^	0.07 ± 0.05	−1.6 ± 0.3	–	–
Tasselled cap wetness (lead)^32^	–	–	0.2 ± 0.08	−0.1 ± 0.21
Tasselled cap wetness^32^	0.22 ± 0.07	1.96 ± 0.44	−0.15 ± 0.08	0.57 ± 0.22
Tasselled cap wetness (lag)^32^	−0.34 ± 0.06	−1.22 ± 0.39	–	–
Temperature suitability index (lead)^35^	0.22 ± 0.02	−0.33 ± 0.1	–	–
Temperature suitability index^35^	−0.00 ± 0.03	−1 ± 0.15	–	–
Temperature suitability index (lag)^35^	−0.06 ± 0.03	0.47 ± 0.15	–	–
Elevation^36^	–	–	0.04 ± 0.05	−0.93 ± 0.13
Accessibility to cities^37^	0.1 ± 0.02	−0.86 ± 0.12	0.13 ± 0.04	−0.38 ± 0.11
Forest cover (yes or no)	−0.22 ± 0.03	−0.46 ± 0.21	−0.16 ± 0.05	0.31 ± 0.13

Forest cover is a binary variable taking the value one if there is no forest and zero if there is.

**Fig. 5:**
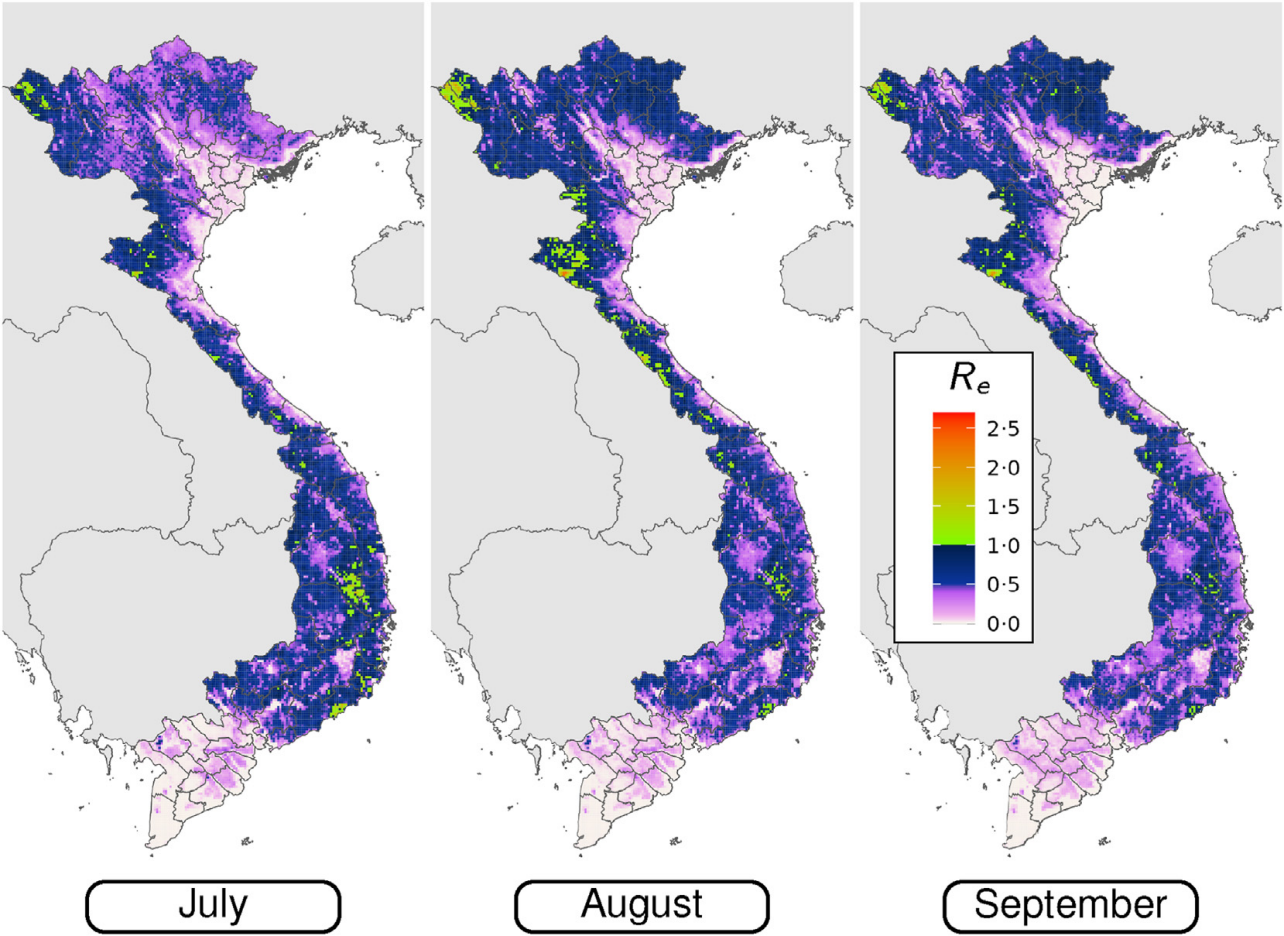
Monthly averages of $R_{e}$ predictions for *P. falciparum* from July to September for the years 2019–2022.

The maps in Fig. 5 illustrate the spatial variation in $R_{e}$ for *P. falciparum* from July until September. During this time period a number of hotspots arise with $R_{e}$ greater than one. These hotspots can be found in the Gia Lai district, along the South Central Coast, and in the north west of the country. These confined pockets represent areas where exponential case growth is possible during these months. The region in Gia Lai is of particular significance, because this is an area with high vulnerability (Fig. 4).

The seasonality of receptivity in Vietnam is not spatially uniform. Regional summaries, which can be found in Figure SI1, illustrate more exaggerated seasonality fluctuations in some regions. The mean $R_{e}$ estimates are all less than one at regional level, indicating that $R_{e}$ is not expected to exceed one over regional length scales under current levels of control. The confidence intervals (incorporating both model uncertainty and within-region variation) around these mean values seasonally exceed one for the Northeast, Northwest, Central Highlands, and South Central Coast regions. During these windows of time, which are closely associated with the wet season,^38^ the risk of outbreaks is higher.

The malariogenic potential illustrated in Fig. 4 highlights the concurrence of vulnerability and receptivity. Given the strong clustering of importations, which lead to hotspots on the vulnerability maps, the malariogenic potential exhibits similar hotspots. The highest malariogenic potential values are observed for *P. falciparum* in the area surrounding the south east of Gia Lai. This area corresponds to the concurrence of high importation rates and high predicted receptivity. 95% Confidence intervals for each of the metrics illustrated in Fig. 4 can be found in Supplementary Material F.

## Discussion

Eliminating malaria from any country requires granular understanding of malaria risk and how it varies geographically and though time. However, measurement of malaria risk becomes increasingly difficult as elimination is approached. Here, for Vietnam, we show how a combination of models can take routine case reporting data and use it to infer crucial—but otherwise hidden—characteristics of the underlying patterns of malaria risk that can provide strategically useful information for eliminating residual malaria transmission and for preventing re-establishment.

Two measures at the core of our analysis are vulnerability and receptivity; hotspots of either quantity pose different impediments to elimination. The combination of both (i.e., the malariogenic potential) summarises the expected combinatorial outcome of importation and local transmission. There is considerable spatial and temporal heterogeneity in the maps we have produced. Such patterns highlight concentrated regions and windows of greater risk. Vulnerability hotspots, such as those illustrated Fig. 4, require interventions tailored towards mitigating the impact of imported cases. These measures could include enhanced surveillance at border areas, health screening for travellers, and public awareness campaigns about malaria prevention to reduce the risk of malaria transmission from imported cases. Windows of time where receptivity is lowest correspond to locations where, under current intervention effort, transmission is more likely to be self-terminating, potentially providing opportunities to target local malaria for elimination.

The time-averaged $R_{e}$ for both parasites, as illustrated in Fig. 4, is less than one throughout Vietnam. This is consistent with the fact that malaria case numbers have continued to decrease in Vietnam over the study period. There is, however, notable seasonal variation in $R_{e}$ for *P. falciparum*. In localised pockets, $R_{e}$ is predicted to exceed one for some of the wet season months, particularly August and September in Gia Lai, the north west, and coastal south east. At such times case numbers may grow exponentially in these pockets. The confinement of residual cases to these more receptive pockets over time means that $R_{e}$ estimates from population-level data, or case-based $R_{e}$ without any spatial context (e.g., Fig. 3), must be interpreted carefully when evaluating progress towards elimination. The average case-based $R_{e}$ does not need to continually decrease to mark progress; in fact, an increase is possible when transmission is eliminated in regions of lower receptivity.

While expected $R_{e}$ values are less than one on larger spatial scales, seasonal windows of $R_{e}>1$ are within the confidence intervals for the Northwest, Central highlands, and South Central Coast regions. So, despite the low average $R_{e}$ values overall, there are spatiotemporal pockets where $R_{e}$ may exceed one, leading to outbreaks if appropriate vigilance is not maintained. Maps such as those presented here can be used to identify regions of elevated risk where increased vigilance is necessary, which is useful for resource allocation and decision making around outbreak preparedness.

Appropriate vigilance for pockets of high predicted $R_{e}$ will depend on whether cases are likely to be imported into these areas. For example, vulnerability is low in the North West region, moderate in the South Central Coast region, and high in Gia Lai. Because of the concurrence of vulnerability and receptivity (quantified as the malariogenic potential), many of the indigenous *P. falciparum* cases have occurred in and around the Krông Pa district of Gia Lai. In contrast, far fewer indigenous cases have been recorded in North West region, despite relatively high receptivity.

The conceptual approach used in this paper was derived from that proposed by Reiner et al. (2015)^22^; however, we computed each metric using updated techniques. For estimating the probability network, we used the more sophisticated diffusion network model of Routledge et al. (2021).^28^ In contrast to the model of Reiner et al. (2015), where the length and time-scales are unknown and “swept over”, this more sophisticated model estimates the parameters by maximising a likelihood function derived from transmission dynamics. To estimate the vulnerability, we used a log-Gaussian Cox model. This model accounts for spatially correlated random effects and does not require the introduction of “background points” (the number and placement of which impact the solution). For mapping $R_{e}$ we used month-specific covariates to capture the climate driven seasonality of receptivity. Moreover, we introduced a novel weighted-regression to adjust for bias due to preferential sampling. The modelling framework used in this paper therefore represents an enhancement to that of Reiner et al. (2015)^22^ that reflects recent methodological advances.

Despite these advances, the models used in this paper are still limited by a range of factors: the reliability of the labels for the observed cases (imported, relapse *etc.*), a detailed understanding of the local drivers of transmission for estimating $R_{e}$, and the availability of data on factors that will influence $R_{e}$. For example, population mobility will factor into the spread of the disease and would therefore be an important addition to the network model and the geospatial model.

Considering the impact of data bias on statistical models is an important component of this modelling pipeline, particularly as observations become sparser in countries approaching elimination. One form of observation bias is the preferential sampling of some areas (and times) over others. This can result from non-uniform importation rate (see Fig. 4) and from the fact that receptivity determines how many subsequent cases will result from a given index case. We have attempted to adjust for this form of bias by weighting each observation inversely proportional to its relative abundance compared to other observations.

A related consequence of receptivity-dependent sample abundance is the need for caution when interpreting time-series such as those in Fig. 3. We did not observe a clear decrease in receptivity over the four-year study window, despite the drop in total case numbers. This is consistent with the idea that regions with low receptivity will produce fewer cases, potentially halting local transmission. As a result the remaining observations will tend to be from pockets of residual local transmission where transmission remains higher.

Another form of observation bias is the complete absence of observations in some areas. This bias cannot be accounted for using the available data alone. This is especially relevant in places where there are no observations, but we have predicted receptivity, such as provinces of the Mekong River Delta and Red River Delta regions. The $R_{e}$ predictions in both of these regions (Fig. 4) are much lower than the rest of the country. In such cases it is important to understand why malaria was eliminated (or not observed), and why it remains so. For example, the Mekong River Delta region benefits from the absence of drug-resistant variants, an ineffective dominant vector species (*Anopheles epiroticus)*, and high levels of access to good healthcare.^39^ This deeper understanding is particularly vital if these explanatory factors are threatened. For example the Mekong River Delta is in close proximity to the Central Highlands region and Cambodia, both of which have high levels of drug-resistant variants^40^ that, if imported, are more difficult to control.

The distinction between local and indigenous cases is fundamental to the analysis in this paper. The current definition of importation is derived from recent travel history and includes travel from other Communes. Including subnational travel in the definition for imported cases is a good first approximation, but introduces two weaknesses in the analysis. If an infection was obtained in one region but observed in another, it may not be accounted for in the estimated $R_{e}$ value of the source region. This is because cases that are too far apart cannot be related under the assumptions we have made about transmission probability and distance. Thus, we are underestimating the receptivity of source regions from which infected individuals leave. The second weakness is that we miss corresponding information on a regional level about sources of importation. A possible modification in future research is to decompose cases with subnational travel history into a susceptible point (which can only be infected) and an infectious point (which can only infect) depending respectively on the suspected location of infection and the location during the period of infectiousness. A more substantial modification would be to incorporate parasite genetic data or human mobility data for improved accuracy when linking cases.^41^^,^^42^

The lack of relapsing *P. vivax* cases in the data set suggests that some of the *P. vivax* cases may be misclassified as new infections. Relapse cases are treated differently to new infections within the diffusion network model, which assumes that the relapse is not a consequence of local transmission but can be a source for local infection. If we have many relapsed cases that are misclassified as indigenous, we will overestimate the receptivity of a region. This misclassification may also conceal seasonal variation in *P. vivax* receptivity, with relapse being falsely considered local transmission in non-receptive months.

The methodologies adopted in this paper are readily adaptable to settings beyond Vietnam. The combination of models not only identifies higher risk areas, but also distinguishes the different dimensions of risk, which can help shape policy and strategic planning. Vulnerability maps, which were estimated using a spatial point-process model trained on importation data, illustrate heterogeneous importation risk. Individual-based estimates, such as those emerging from the diffusion network model, can provide enhanced insight over population-level statistics. These metrics will become even more vital as population level measures become less relevant near elimination. All endemic countries need to transition through a non-endemic phase before being declared malaria free. The techniques and analysis presented in this paper can support this transition.

This analysis can be extended to the entire GMS by incorporating case data from the other GMS countries, which would allow for a more holistic approach to risk quantification and support elimination goals in the sub-region. A GMS-wide analysis could be used to quantify transnational barriers to elimination rather than the country specific challenges, which may not factor in the risks posed by malaria in neighbouring countries. Being able to probabilistically link cases of malaria makes the diffusion-network model distinctly suited for a cross-border analysis of transmission, which is an important topic for the elimination of malaria in the GMS and beyond.^43^^,^^44^ Furthermore, a GMS-wide analysis would be able to better account for international importation and exportation of *Plasmodium*, which is critical for understanding the spread of resistance to antimalarial drugs within the GMS.^45^

The results presented here provide new and strategically useful insights into receptivity and vulnerability to importation of *P. falciparum* and *P. vivax* malaria in Vietnam; metrics that represent distinct components of malaria risk. Building on past research, important methodological advances were developed for modelling malariogenic potential in near-elimination settings, which are potentially applicable in many malaria endemic countries. Such techniques will become critical when countries face new challenges as they progress from near-elimination to malaria-free. As Vietnam's stated target year for elimination of 2030 rapidly approaches, the techniques and outputs presented here may usefully inform policy and help accelerate progress toward achieving this important national public health goal.

## Contributors

MM contributed to formal analysis, investigation, methodology, software, validation, visualisation, writing-original draft, writing-review and editing. YAG contributed to conceptualisation, formal analysis, data curation, funding acquisition, investigation, methodology, project administration, validation, writing-original draft, writing-review and editing. NXT, NTHP, and NDT contributed to data curation, writing-review and editing. WHO contributed to conceptualisation, data curation, funding acquisition, Writing-review and editing. FJIF and PWG contributed to conceptualisation, funding acquisition, writing-review and editing. TLS contributed to methodology, writing-review and editing. DJW contributed to writing-review and editing. This data has been accessed and verified by Dr Michael McPhail and Dr Yalemzewod Gelaw. Both Dr Michael McPhail and Dr Yalemzewod Gelaw are responsible for the decision to submit the manuscript.

## Data sharing statement

The deidentified case data for this study was provided by the National Institute of Malariology, Parasitology and Entomology (NIMPE), Ministry of Health, Hanoi, Vietnam. The data cannot be made publicly available. Readers who are interested in using this data should contact NIMPE directly. A representative data set, which is not based on real case data but can be used to test the code base, can be found in the code repository described below.

## Code availability

The code for the analysis in this paper are available from https://github.com/michaelmcphail/malaria_metrics_near_elimination.

Archived analysis code can be accessed from https://doi.org/10.5281/zenodo.14603382.

## Editor note

The Lancet Group takes a neutral position with respect to territorial claims in published maps and institutional affiliations.

## Declaration of interests

The authors declare no competing interests.
