# Supplementary material for: Mapping residual malaria transmission in Vietnam
Source: Lancet Reg Health West Pac. 2025 Apr 10;57:101545. doi: 10.1016/j.lanwpc.2025.101545 (PMC12008676; doi:10.1016/j.lanwpc.2025.101545)
Supplement: Supplementary Material [file mmc1.pdf]

Supplementary Information

February 24, 2025

Contents

A Supplementary figures **2**

B Network diffusion model **3**
B.1 Key ideas of the network diffusion model . . . 3
B.2 A network model . . . 4

C Log-Gaussian Cox model **5**
C.1 On the spatial structure of imported cases . . . 6

D Sampling bias **7**
D.1 Statement of problem . . . 7
D.2 Adjusting for sampling bias . . . 10
D.2.1 An illustrative example . . . 10
D.3 A proposed solution . . . 12
D.4 Simulation framework . . . 13
D.4.1 Key findings . . . 14
D.5 Testing this approach on real data . . . 15

E Geospatial model for R_e **15**

F Confidence intervals and sensitivity analysis **17**
F.1 Confidence intervals . . . 17
F.2 Sensitivity analysis . . . 18
F.2.1 What if all cases are local? . . . 18

A Supplementary figures

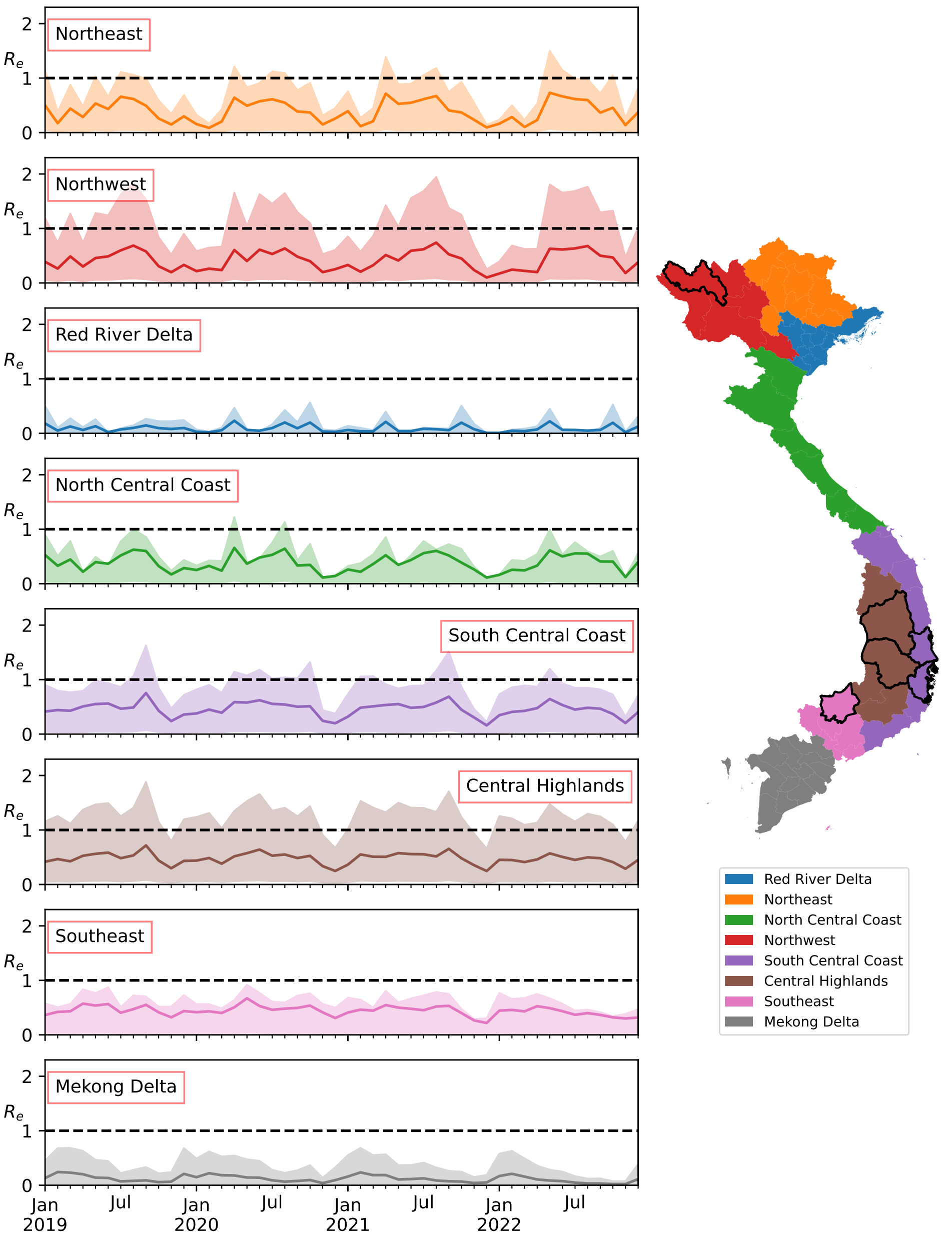

Figure A.1: Zonal summaries of monthly population-weighted mean R_e predictions for P. falciparum in each region. Solid lines indicate expected values and shaded areas indicate the 95% confidence interval.

B Network diffusion model

In this supplementary section we detail the network diffusion model for inferring transmission probabilities. The methods described here are not novel; we only summarise descriptions given in existing literature and include only the specifics of the method relevant to the paper. A complete description of the technical details contained in this section can be found in work by Gomez-Rodriguez *et al.* [2, 3]. The malaria-specific components can be found in Routledge *et al.* [6, 7, 8].

B.1 Key ideas of the network diffusion model

A common data format in near elimination settings is *line-list* data, which contains time-stamped individual malaria cases. Sometimes this data is geolocated, at least to the health facility level. To better understand the epidemiological factors that gave rise to these observed cases, it would be useful to know how the cases were linked by transmission. The transmission network is difficult to measure directly, and would require genetic sequencing to know for sure. Ideally, we could use the space-time coordinates of the cases to infer the the chains of transmission; that is, we would know which individual was the source/infector of each case. With many cases occurring in close proximity (in space and time), it is unrealistic to expect that we could infer this information from time-of-onset and location data alone. Instead, the next best outcome would be a *probability network*, where nodes represent cases and weighted, directed edges indicate the probability that a source node infected a target node. Inferring this probability network from the observed cases is the motivation behind using the network diffusion model.

Suppose that the input data comprises N cases and assume that we know the location and time (of symptom onset) for each case. We represent this data as the set of coordinates $\mathbf{t} = \{(\mathbf{x}_1, t_1), ..., (\mathbf{x}_N, t_N)\}$, where $\mathbf{x}_i$ and t_i are the respective location and time of case i. We order the cases in time-order, such that $t_i \geq t_{i-1}$.

A *cascade* is a sub-graph of a network where nodes of the cascade are infections, and edges represent the source of infection. In this case we aren't directly observing the the cascade, but instead the activation times and locations of nodes as the contagion propagates across the network. There may be multiple cascades that would result in a single observation $\mathbf{t}$. This family of cascades represents all possible transmission pathways that could explain $\mathbf{t}$. To infer the underlying network, given $\mathbf{t}$, we impart structure on the possible relationships between cases using the mechanisms of transmission. This structure limits can be used to prohibit some edges based on *plausibility* and to assign the remaining edge-weights based on *probability*.

We assume that cases can only be the source of infection for other cases that occur sufficiently later in time. Specifically, case j can only be the source of infection for i if the time between symptom onset is at least $\gamma = 15$ days. This period of delay corresponds to the incubation period between an infectious bite and the onset of symptoms in i. We assume that a case i can only have one parent; neglecting multiclonal infections. The conditional likelihood of transmission from j to i, $f((\mathbf{x}_i, t_i)|(\mathbf{x}_j, t_j), \boldsymbol{\theta}_{i,j})$ parameterised by $\boldsymbol{\theta}_{i,j}$, is a fundamental determinant of the network structure that will emerge using this approach. A simple model would be to assume that the likelihood depends on the proximity between cases (in space and time). More sophisticated likelihood parameterisations might use a more abstract notion of proximity between cases by factoring in population movement between locations and socio-demographic factors.

Following [8], we use a shifted Rayleigh distribution to parameterise the shape of the transmission likelihood in time. We also assume that the spatial and temporal components of the likelihood are separable[1] and assume that the distance component of the transmission likelihood follows a Gaussian distribution. The subsequent likelihood function for pairwise infections is given by

$$f((\mathbf{x}_i, t_i)|(\mathbf{x}_j, t_j), \alpha_{i,j}, \beta) = \alpha(t_i - t_j - \gamma) \exp\left[-\frac{1}{2}\alpha_{i,j}(t_i - t_j - \gamma)^2 - \beta(\mathbf{x}_i - \mathbf{x}_j)^2\right], \tag{B.1}$$

where $\alpha_{i,j}$ is the interaction-specific temporal rate parameter, and β parameterises the decay of transmission likelihood with distance.

[1] Whether or not this holds will depend on the details of transmission.

The following three standard definitions make the statement of network likelihood easier to interpret. The cumulative density function (evaluated at t_i and $\mathbf{x}_i$), given by[2]

$$F((\mathbf{x}_i, t_i)|(\mathbf{x}_j, t_j), \alpha_{i,j}, \beta) = \int_{t_j}^{t_i} \int_0^{\infty} f((\mathbf{x}, t)|(\mathbf{x}_j, t_j), \alpha_{i,j}, \beta) \mathrm{d}x \mathrm{d}t, \tag{B.2}$$

is the probability that j could activate i at any time between t_j and t_i. Conversely, the probability that i survives it's interaction with j (*i.e.*, remains uninfected) until t_i is known as the *survival function*, S, and is given by

$$S((\mathbf{x}_i, t_i)|(\mathbf{x}_j, t_j), \alpha_{i,j}, \beta) = 1 - F((\mathbf{x}_i, t_i)|(\mathbf{x}_j, t_j), \alpha_{i,j}, \beta). \tag{B.3}$$

Specifically, the survival function is the probability that node i is not activated by node j until at least t_i. Finally, the instantaneous rate of infection between j and i is known as the *hazard function*, H, and is given by

$$H((\mathbf{x}_i, t_i)|(\mathbf{x}_j, t_j), \alpha_{i,j}, \beta) = \frac{f((\mathbf{x}_i, t_i)|(\mathbf{x}_j, t_j), \alpha_{i,j}, \beta)}{S((\mathbf{x}_i, t_i)|(\mathbf{x}_j, t_j), \alpha_{i,j}, \beta)} = -\frac{S_t((\mathbf{x}_i, t_i)|(\mathbf{x}_j, t_j), \alpha_{i,j}, \beta)}{S((\mathbf{x}_i, t_i)|(\mathbf{x}_j, t_j), \alpha_{i,j}, \beta)}. \tag{B.4}$$

We've included the right-most side of the equation here to illustrate that link between the hazard function, the survival function and the rate of change in time of the survival function, S_t. Specifically, the hazard function can be expressed as $H = -\mathrm{d}(\log(S))/\mathrm{d}t$.

B.2 A network model

The quantities introduced and the assumptions made in §B.1 all relate to individual interactions between cases. The next step is to calculate the subsequent probabilities of interactions in the context of all other possible interactions. The steps involved in this calculation can be found in [2], and will not be repeated in detail here. Instead we will discuss the important dynamics factored into this calculation, and then present the final result.

The survival function (B.3) specifies the probability that i *survives* it's interaction with case j such that it only activates at t_i and no earlier. Given the multitude of cases, in order to only activate at t_i, case i must survive all interactions with plausible sources until t_i. Thus, for j to be the source of infection of i, and for i to activate at t_i, i must survive all interactions with other cases and survive the interaction with j for long enough to only activate at t_i. The likelihood of this happening will increase if the survival functions for all interactions are large, and if the hazard function for j to i at t_i is large.

Factoring in these transmission likelihoods as well as the competition between possible sources to be the infector of i, we get a likelihood function for an observation $\mathbf{t}$ given by

$$f(\mathbf{t}|\mathbf{A}, \beta) = \prod_{t_i} \prod_{k:t_k<t_i} S(t_i|t_k, \alpha_{i,k}, \beta) \sum_{j:t_j<t_i} H(t_i|t_j, \alpha_{i,k}, \beta), \tag{B.5}$$

where have we dropped reference to the spatial coordinates for readability. The most likely graph, given the observations $\mathbf{t}$, can therefore be found by solving the following optimisation problem

$$\mathrm{argmin}_{\boldsymbol{\alpha},\beta} \left[-f(\mathbf{t}|\mathbf{A}, \beta)\right], \tag{B.6}$$

subject to $\alpha_{i,j} \geq 0$ and $\beta > 0$. Solving this optimisation problem is the objective of the `NetRate` algorithm described in [2]. We use an algorithm based on the code provided by Routledge *et al.*, [7] to achieve this.

We incorporate prior information on $\alpha_{i,j}$ and β by Baye's theorem. Our prior distributions for both sets of hyperparameters, $\alpha_{i,j}$ and β, are truncated normal distributions given by

$$f(\theta|\mu, \sigma, a, b) = \frac{1}{\sigma} \frac{\phi\left(\frac{\theta-\mu}{\sigma}\right)}{\Phi\left(\frac{b-\mu}{\sigma}\right) - \Phi\left(\frac{a-\mu}{\sigma}\right)} \tag{B.7}$$

[2]Note that this is where our assumption of space-time separability becomes significant. The assumption here is that the distance between points is Gaussian distributed, and completely independent of time. Thus, this integral states that at every *slice* of time, we integrate over all possible points.

for $a \leq \theta \leq b$ and zero otherwise. Here μ is the prior mean, σ is the prior standard deviation, a is the lower bound, and b is the upper bound. The standard normal distribution, $\phi(x)$, is given by

$$\phi(x) = \frac{1}{\sqrt{2}} \exp\left(-\frac{x^2}{2}\right) \tag{B.8}$$

and the standard cumulative distribution function $\Phi(x)$ is given by

$$\Phi(x) = \frac{1}{2}\left(1 + \mathrm{erf}\left(\frac{x}{\sqrt{2}}\right)\right). \tag{B.9}$$

We take $a = 0$ because if $\alpha_{i,j}$ (or β) is less than zero, the likelihood of infection increases exponentially (or super exponentially) with time (or distance), which is physically implausible. The upper bound of $b = 1$ sets a precision threshold beyond which the likelihood of interaction cannot be distinguished. That is, if b were to take values greater than 1, then the lengthscale of transmission would be less than approximately 1km (and the model would find almost none of the cases in the data set can be related by transmission). We assume a prior mean on α of $0.003\,\text{days}^{-1/2}$ and standard deviation of $0.001\,\text{days}^{-1/2}$. This corresponds to a peak in the Rayleigh distribution (the serial interval) used to parameterise the interaction likelihood (B.1) of 33 days. We assume a prior mean on β of $0.001\,\text{km}^{-1/2}$ and standard deviation of $0.001\,\text{km}^{-1/2}$. This corresponds to a mean interaction lengthscale of 31 km.

What we haven't mentioned here is the possibility of unobserved sources of infection; that is, infectious cases that aren't contained in our data, but are responsible for infection. The augmentation of (B.5) to include unobserved cases is straightforward, and detailed in Routledge *et al.*, [8]. In practice, we prescribe the survival and hazard functions for node i to an unobserved source to be given by

$$S_0(t_i|\epsilon) = \exp(-\epsilon), \qquad H_0(t_i|\epsilon) = \epsilon, \tag{B.10}$$

where ϵ relates to the force of infection from any potential unobserved source. We have very little information about the influence of unobserved sources, which will depend on factors outside of the available data. Typically, ϵ is taken to be small enough that an unobserved source is only invoked if none of the observed cases could plausibly have infected i under the assumed model of transmission. However, if you have sufficient prior information of flaws in the surveillance system corresponding to many unobserved sources, then the prior distribution for ϵ can be assumed to have larger mean value.

C Log-Gaussian Cox model

Reiner *et al.*[5] estimate vulnerability using a logistic regression with a general additive model (GAM). This approach requires sampling *background points*; points where there are no observed importations. It is unclear, however, the appropriate number of background points needed or where they should be sampled. A more natural interpretation of importation events can be constructed from a Poisson point process.

A point pattern, Y, from a Poisson point process on domain Ω has likelihood, π, given by

$$\pi(Y|\lambda) = \exp\left[|\Omega| - \int_{\Omega} \lambda(s)\mathrm{d}s\right] \prod_{s_i \in Y} \lambda(s_i), \tag{C.1}$$

where $|\Omega|$ is the area of the domain. The intensity of the point process is denoted λ, and the expected number of points in some sub-region $\hat{\Omega} \subset \Omega$ is given by

$$\mathbb{E}\left[N(\hat{\Omega})\right] = \int_{\hat{\Omega}} \lambda(s)\mathrm{d}s. \tag{C.2}$$

For a log-Gaussian Cox process (LGCP) model, the intensity is given by $\log(\lambda(s)) = Z(s)$, where $Z(s)$ is a Gaussian random field. Thus, for a LGCP the log-likelihood of Y for an intensity

surface λ is given by

$$\log\left[\pi(Y|\lambda)\right] = |\Omega| - \int_{\Omega} \exp\left[Z(s)\right] \mathrm{d}s + \sum_{i=1}^{N} Z(s_i). \tag{C.3}$$

The last two terms on the right hand side of (C.3) depend on the random field $Z(s)$, and only the last term depends on the point pattern Y.

Optimising the random field parameters against the log-likelihood (C.3) is challenging. The domain must be discretised, and a discrete representation of the random field constructed. A common approach is to use a fine regular lattice to discretise the domain. The number of observations in each cell is computed, which are independent Poisson random variables. The intensity of the Poisson process is approximated by $\Lambda_{ij} \approx |s_{ij}| \exp(z_{ij})$. So, the finite-lattice approach determines the parameters by fitting the Poisson intensities to the observed number of observations in each cell. Sampling from the random field is computationally expensive as the multivariate Gaussian vector $\mathbf{z}$ has a dense covariance matrix. The subsequent system can be drastically simplified by using a conditional auto-regressive model on the fine lattice as a discrete approximation to the random field. A downside of this approach is that the lattice now serves a dual purpose: it supports the random field and bins the observations. We must trade-off between a lattice sufficiently fine to resolve spatial patterns in the observations, but without too many cells to make fitting for for the random field intractable.

A more tractable approach is to use a finite-dimensional representation of a random field. For more see Simpson *et al.*[9]. Let the random field, $Z(s)$, be given by

$$Z(s) = \sum_{i=1}^{n} z_i \phi_i(s), \tag{C.4}$$

where z is a multivariate Gaussian random vector and $\{\phi_i(s)\}_{i=1,\dots n}$ is a set of linearly independent basis functions. In this approach, $Z(s)$ is spatially continuous. So, to evaluate the likelihood (C.3) we choose an appropriate discrete approximation to the integral (as opposed to spatially discretising the field and then approximating the integral). The updated log-likelihood of (C.3) is now expressed in terms of the basis functions and integration weights α as

$$\log\left[\pi(Y|\lambda)\right] \cong |\Omega| - \sum_{i=1}^{p} \alpha_i \exp\left[\sum_{j=1}^{n} z_i \phi_i(s)\right] \mathrm{d}s + \sum_{i=1}^{N} \sum_{j=1}^{n} z_i \phi_i(s_i). \tag{C.5}$$

The evaluation of the likelihood (C.5) can be performed using the `INLA` library in R.

We employ a model of the form

$$y \sim b_0 + Z(s). \tag{C.6}$$

We have two sets of points to consider: the location of the mesh nodes and the locations of importations. We specify observations and weights at both of these sets of points. The target variable is given by $y = [\{0\}_{i=1,\dots,n_{mesh}}; \{1\}_{i=1,\dots,n_{obs}}]$. We also specify the known component, E, of the Poisson likelihood $E\exp(\lambda)$ to be given by $E = [\{\alpha_i\}_{i=1,\dots,n_{mesh}}; \{0\}_{i=1,\dots,n_{obs}}]$.

C.1 On the spatial structure of imported cases

The initial choice to use a log-Gaussian Cox model was based on the type of data (a point pattern), the desired output (a model to describe importations), and the apparent spatial clustering of the data (as illustrated in figures C.1a and C.2a). The log-Gaussian Cox process model is a flexible approach, and the intensity fields illustrated in figures 4 and 5 of the main text seem to capture the areas where importation is important, but it is still important to verify that this model is appropriate. In this section we examine some of the assumptions underlying the use of a log-Gaussian Cox model.

We can get some indication if the LGCP is appropriate for the observed data by studying an nonparametric intensity approximation using kernel density estimation (KDE). Using a KDE we

can infer two things: whether or not the Gaussian process model could explain the observations (do we see smooth variations in the log-intensity?), and whether or not spatial correlation is appropriate for a Gaussian process (by analysis of the semivariogram).

The log-intensity of the KDE is illustrated in Figures C.1a and C.2a for P.*falciparum* and P.*vivax* respectively. In neither case does the log-intensity display sharp variation anywhere in the domain; such a function is readily ammenable to approximation by a Gaussian process. The empirical variogram of the log-intensities are illustrated in illustrated in Figures C.1a and C.2a for the respective parasites. Both variograms illustrate the characteristic structure expected in a spatially correlated random process; namely, a range, which indicates the lengthscale of correlation, and a sill, which indicates the plateau. Both of these pieces of evidence indicate that a LGCP is a viable model to describe the spatial point pattern.

To further motivate a spatially inhomogeneous point process model, rather than a spatially stationary model, we use Ripley's K-function, which is given by

$$K(r) = \frac{a}{n(n-1)} \sum_i \sum_j \mathcal{I}(d_{ij} \leq r) e_{ij}, \tag{C.7}$$

where a is the domain area, n is the number of data points, $\mathcal{I}$ is an indicator function (equal to 1 if the distance between points i and j is less than r, and zero otherwise), and e_{ij} is an edge correction weight. The K-function is the expected number of points within some radius For a spatially stationary (Poisson) point process $\hat{K}(r) = \pi r^2$. $K(r)$, for the observed points, greater than $\hat{K}(r)$ is suggestive of spatial clustering. That is, the number of points surrounding a given observation is greater than would otherwise be expected if the intensity was spatially uniform.

We plot the Ripley's k-function for P.*falciparum* and P.*vivax* in Figures C.1c and C.2c respectively. We note that for both patterns, the k-function is substantially above $\hat{K}(r)$, suggesting strong spatial clustering. Caution is required, however, because the k-function should be monotonic. In both cases, there is non-monotoniticy for higher r values. This is likely due to how edge effects are handled in the computation of the k-function, and the fact that many imported cases are closer to boundaries than they are other points. So, the k-function will only be valid for relatively small distances r. There is clear evidence for clustering, however, even over small distances.

D Sampling bias

D.1 Statement of problem

As countries approach elimination observation bias will make statistical inference, particularly geostatistical inference, increasingly difficult. With fewer recorded cases there is less data to inform statistical estimates. Not only will there be fewer observations, but the spatial and temporal coverage of these cases will become less comprehensive, and possibly skewed to epidemiologically unusual pockets. There are a number of reasons for heterogeneous observation distribution, some of which depend on the quantities we are attempting to estimate from the data.

One source of sampling bias is that residual transmission is more likely to occur in difficult to eliminate locations. Another is that temporal variation in receptivity (*e.g.*, reproduction number), for example through seasonal variation, can lead to a greater concentration of observations during certain times of the year and a sparsity otherwise. In both of these examples the abundance of observations depends on the receptivity; the quantity we are trying to estimate.

Another source of bias arises through non-uniform case importation. Imported cases can initiate transmission chains (or transmission trees if all descendants are linked to the imported index case), and for the purposes of statistical modelling represent sample sites. The resultant transmission reflects the receptivity of that location for the duration of the outbreak. The ideal scenario for statistical inference would be to have plentiful, uniform importation[3]. In practice importation rate is spatially heterogeneous, so the subsequent observations are preferentially sampled in regions with greater importation.

To train a statistical model for receptivity (measured by R_e) we must account for two independent sources of sampling bias. The first is spatially heterogeneous case importation, the pattern of

[3] This would be non-ideal from the perspective of malaria control

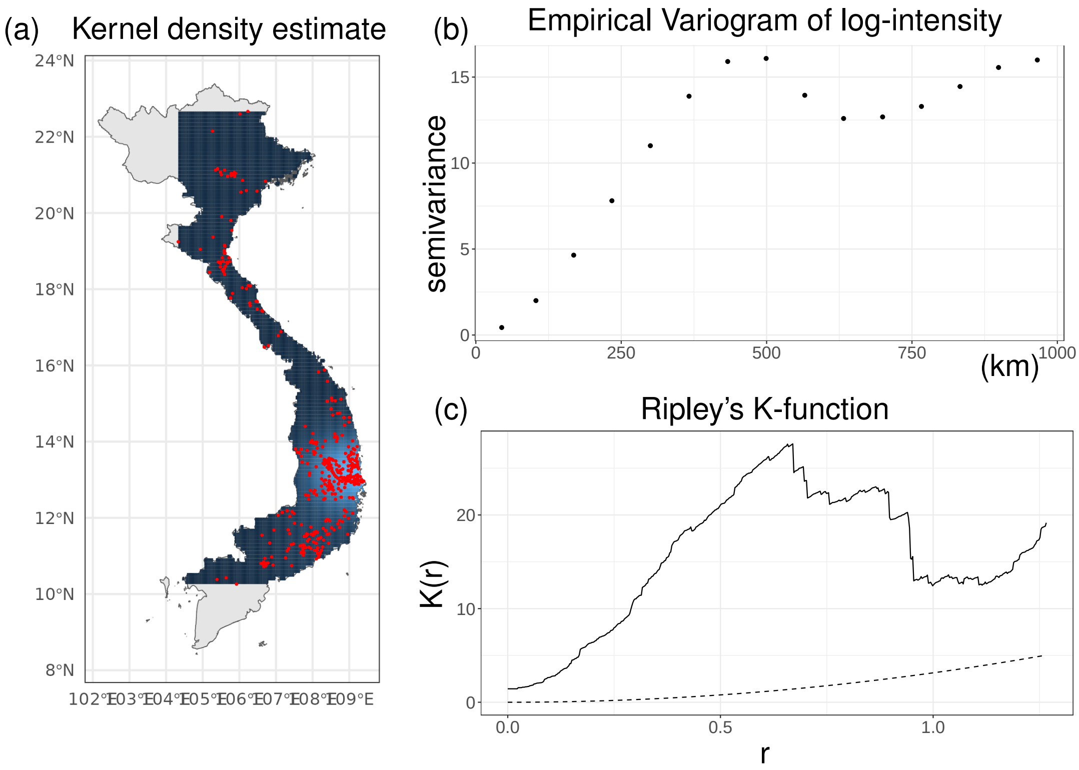

Figure C.1: For the imported cases of P.*falciparum* we illustrate (a) the Kernel Density Estimate (KDE) for the point pattern illustrated by the red points; (b) the empirical variogram computed from the KDE; and (c) Ripley's K function illustrating the expected number of points within a distance r of another.

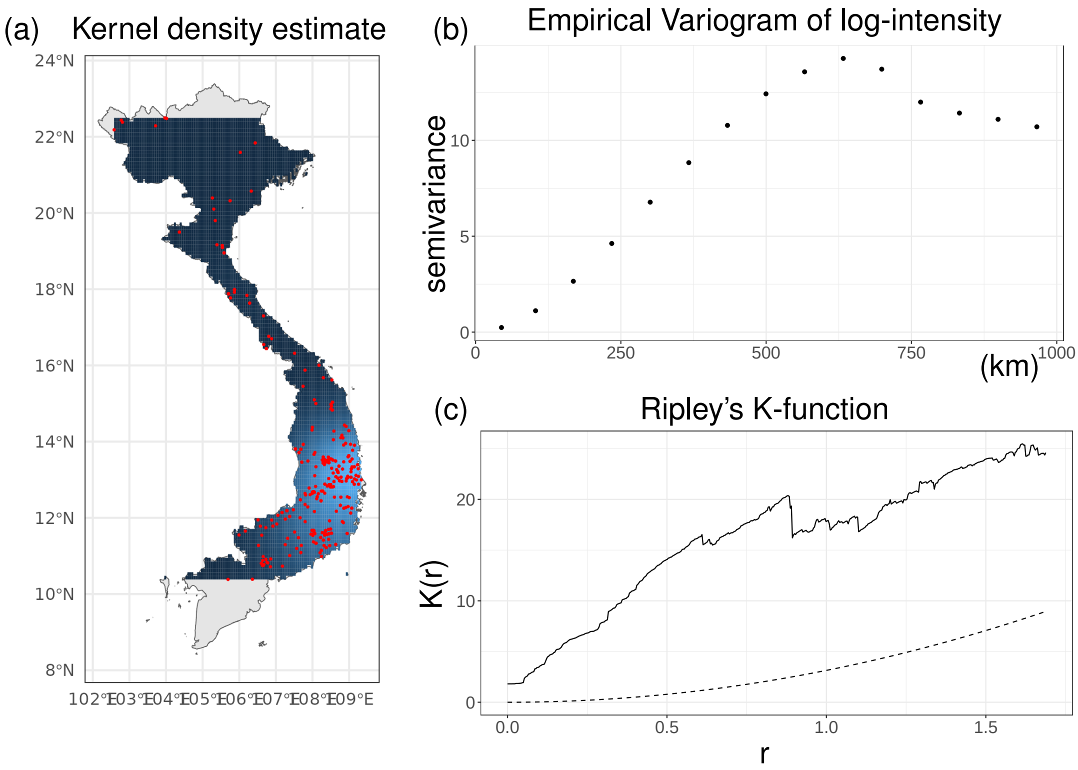

Figure C.2: For the imported cases of P. *vivax* we illustrate (a) the Kernel Density Estimate (KDE) for the point pattern illustrated by the red points; (b) the empirical variogram computed from the KDE; and (c) Ripley's K function illustrating the expected number of points within a distance r of another.

which is independent of the target variable R_e. The second source of bias is the fact that pockets (with limited spatial or temporal extent) of greater R_e generate more cases. This source of bias is dependent on the target variable of our analysis.

Not accounting for different rates of observation at different places and times will lead to biased parameter estimates. Birello *et al.* [1] demonstrate that population level estimates of reproduction number may be biased when there is underlying spatially structured populations. Moreover, they demonstrate that weighting the community-level incidence counts appropriately can correct for this bias. While the population-level analysis of Birello *et al.* is fundamentally different to the individual-based calculation of this paper, one of the core features is the same: not accounting for heterogeneous transmission dynamics can introduce bias. Thus, the bias we attempt to correct for is not solely a consequence of being in a low-data setting. The influence of the reproduction number on the number of observations will introduce correlated observations at all levels of transmission.

D.2 Adjusting for sampling bias

One approach for adjusting for the sampling biases is to use a weighted likelihood method. In this case, we weight the contribution of each observation to the likelihood function. Weights are chosen to de-emphasise some observations and give more prominence to others. Normally, if some data is less representative of the underlying process (*i.e.* potentially compromised) we would use smaller weight values for those data values. In our case we wish to down-weight over-represented data.

When deciding whether it is appropriate to use a weighted regression method, we must consider the variance–bias trade off. Data with higher associated weight will exert more influence on the model. In other words, when non-uniform weights are used the model is more influenced by fewer data points. When all weights are equal (*i.e.*, unweighted), we obtain the lowest variance but expect the highest bias. Conversely, when sampling bias is completely adjusted for using weights we expect an unbiased solution with the largest variance.

We could, if desired, use a continuously varying map between the weighted method and the unweighted method. For example, if the appropriate vector of weights is $\mathbf{w}$, then the weights given by $\mathbf{w}^p$, where $p \in [0, 1]$ can be used to transition between the weighted and unweighted methods. When $p = 1$ we have the weighted method and when $p = 0$ we have the unweighted method. Alternatively, we could use truncate the weights between the $x\%$ and $100 - x\%$ quantiles. When $x = 50\%$ all of the weights are equal.

D.2.1 An illustrative example

Consider a very simple illustrative example. In this example we suppose that the reproductive number R_e varies through time. We observe a case on day $d = 0$ and know that this case infects two other cases, which are both observed on day $d = 1$ and infect no further people. The reproduction number on $d = 0$ is therefore $R_e(0) = 2$ and on day $d = 1$ we have $R_e = 0$. If we don't know the form of the underlying data-generating process[4] we may wish to assume a constant R_e over the time-period. An unbiased estimate of the average reproduction number over the time interval is $\bar{R}_e = 1$, which is the average of the reproduction number over both days. Using linear regression (with only an intercept, no covariate slope) gives the estimate $\bar{R}_e = 2/3$; the average of the three observations. This bias arises because the errors are not independent, which is a key assumption for ordinary linear regression[5].

In this simple example we note that the introduced bias is not a result of having insufficient of data, and will not be mitigated by having more data. Instead, it is a direct consequence of the dynamics. In this situation, for every case observed on day $d = 0$, we will have twice as many cases on $d = 1$. Even if we had more observations on $d = 0$, this relative abundance would always remain.

[4] as is often the case in practice

[5] Note that if we instead asked what the average reproduction number is for an individual case, then $\bar{R}_e = 2/3$ is appropriate. The difference between the questions is somewhat nuanced. The latter (average R_e of the cases) is more dependent to the sampling process (abundance of observations) and, therefore, what might be observed in practice under the same (stationary) conditions. The former (average R_e of that time-window) is more closely tied to the model for R_e. Consider the difference in estimates if N new cases were imported on $d = 1$. Now the case-averaged $\bar{R}_e = 2/(3 + N)$, but the model by which R_e is determined has remained unchanged

We can extend this very simple example. Suppose we have a large outbreak of N observations over some time period, where all observed cases are linked to the index case through a known transmission chain. The mean value of R_e over the time interval is given by

$$\bar{\beta} = \frac{1}{M}\sum_{i=1}^{M} y_i, \tag{D.1}$$

where M is the number of days for which we have observations, and y_i is the value of R_e on day i. An estimate for $\bar{\beta}$ is the solution to a weighted-least-squares regression (with no slope, only an intercept), which is given by

$$\beta = \left(X^T W X\right)^{-1} X^T W \mathbf{y}, \tag{D.2}$$

where $X = (1, ..., 1)_N$ and W is a diagonal matrix containing the observation weights; that is, $\mathrm{diag(W)} = \{\mathrm{w_i}\}_{\mathrm{i=1,...,N}}$ where weights are strictly positive. We will represent the vector of N weights by $\mathbf{w}$. Equation (D.2) simplifies to

$$\beta = \frac{1}{||\mathbf{w}||_1}\sum_{i=1}^{M}\sum_{j=1}^{N_i} w_j y_j, \tag{D.3}$$

$$= \frac{1}{||\mathbf{w}||_1}\sum_{i=1}^{M} N_i w_i y_i, \tag{D.4}$$

where N_m is the number of observations on day m and the last line follows if all observations on day m are equal and equally weighted[6]. We have used the notation $||\cdot||_1$ to denote the 1-norm; i.e., $||\mathbf{w}||_1 = \sum_i |w_i|$. We can arbitrarily choose to scale the weighting vector such that $||\mathbf{w}||_1 = 1$. By comparing the coefficients of y_i in equations (D.1) and (D.4), we see that $\hat{\beta} = \beta$ for all y_i values if and only if we set

$$w_i = \frac{1}{MN_i}. \tag{D.5}$$

By choosing the appropriate weights we can account for sampling bias to remove the estimate bias in this simple toy problem.

Here we've made an implicit assumption that the underlying *true* model that determines R_e is only time dependent; that is, all observations on a given day have perfectly correlated residuals. We can relax this assumption by replacing "days" by "cluster", where cases belong to the same cluster if they have the same ancestry (*i.e.*, were infected from the same source). Thus, if there is some latent field modifying the distribution of R_e values by, say, location then these aren't binned with observations from a different distribution. The arithmetic is more difficult in this case, but still tractable.

For a single outbreak we want to link the relative weight of each observation back to the index case (the infection at the top of the tree). The children of the index case each have children of their own, potentially swamping the information from the observed index case if weighting isn't accounted for. The key idea is to down-weight members of each cluster so that the relative size of the cluster's weight's are $1/N_i$'th the size of the cluster's parent's weight (where N_i is the size of the cluster). This procedure is described in more detail in the next section.

The next extension to this illustrative example would be to consider multiple outbreaks. If outbreaks are seeded at a rate dependent on some probability density across time, the relative likelihood of an outbreak starting at between t and $t + \delta t$ is proportional to the integral of the probability density function over this time interval. So, more outbreaks are seeded where the probability density function is higher. The same over-counting arithmetic applied to local cases can be applied here; that is, if we don't use the appropriate abundance-based weights in the regression, we will calculate biased estimators. In this case the weights are inversely proportional to the probability density function.

[6] This makes the arithmetic simpler, but is not a necessary assumption. If the observations on a given day are not fixed, but drawn from the same distribution, we would calculate the expected value of β and get the same result.

D.3 A proposed solution

To return to the original problem, we have sampling biased introduced by non-uniform outbreak seeding and a receptivity dependent data-generating process. To adjust for sampling bias we propose weighted regression. Building on the ideas discussed in §D.2.1, we construct an algorithm for calculating the weights that first depends whether or not the case has a known parent. If the case has no known parent, the weight is inversely proportional to the rate at which such cases are observed at that location. We estimate this rate using a log-Gaussian Cox model, which is described in the main text and in Appendix C. So, for an orphan case occurring at coordinate $\mathbf{x}$, the weight is given by

$$w(\mathbf{x}) = \frac{C}{\lambda(\mathbf{x})}, \tag{D.6}$$

where C is a normalising constant, and λ is the estimated Poisson intensity, which is necessarily non-zero where there are imported cases.

If the case has a known parent, the weight should be proportional to the product of the weight assigned to the parent and the inverse of the number of children produced by the parent. To illustrate this, consider an index case (grand parent) with two children (parents) who each have 2 and 3 children respectively. There are 5 grand children, but the grand children's residuals will be correlated within groups of 2 and 3 respectively (*i.e.*, amongst siblings). So the grand parent's regression-weight is inversely proportional to importation intensity, the parents weights are half of this, and the grand-children's are 1/4'th and 1/6'th of the grand-parent's weight depending on their parent.

Generally, the weight of some i'th generation case is given by

$$w_i = \frac{w_{i-1}}{(R_e)_{i-1}}, \tag{D.7}$$

where w_{i-1} is the weight of the parent and $(R_e)_{i-1}$ the parent's reproduction number (number of offspring). If the chain of transmission is known, this can be recursively linked to the weight of the index case, giving

$$w_i = \frac{C}{\lambda(\mathbf{x}_0)\prod_{j=0}^{i-1}(R_e)_j}, \tag{D.8}$$

where $\mathbf{x}_0$ is the location of the index case.

In the real-world example discussed in the main text of this paper we don't know the chain of transmission between index cases and offspring. Instead, we have a probability network where edges indicate the probability that two cases are linked by transmission. One option for calculating weights using this information would be to generate a large number of realisations of this probability network, where for each realisation we have a distinct transmission network and can therefore calculate the weights. The weights derived from the probability network are then the mean over these realisations.

A more computationally efficient way to calculate these expected values is to incorporate the probabilities directly into the computation of the weights. So, the weight inherited by a case with multiple possible sources is linked to the probability assigned to each source. Here we are exploiting the conditional independence between most nodes of the network. The chain of transmission prior to the parent doesn't matter, all that matters is the probability of each candidate parent being the parent. The expected weight (*i.e.*, the update to (D.7) given uncertain ancestry) is given by

$$w_i = \left(\sum_{j\in J}\frac{\mathbb{P}_j(R_e)_j}{w_j}\right)^{-1}, \tag{D.9}$$

where J is the set of possible parent nodes, and $\mathbb{P}_j$ is the probability that j is the parent of i.

D.4 Simulation framework

In this section we consider a simplified problem to demonstrate the need to account for observation bias. We develop a simulation framework flexible enough to study the sensitivity of our results to the assumptions under which the data is generated. In this simplified problem we simulate cases using by a prescribed infection process throughout a single year.

In this problem there is no spatial dimension, only time. Cases can occur on any given day between $t = 1$ and $t = 365$. We will specify when cases are imported (index cases). These are cases that can start transmission chains, but they themselves are not the result of local transmission dynamics. We will use a model for that assumes R_e is linearly proportional to some environmental variable that varies throughout the year. Thus, the time of year that a case occurs determines the number of new cases that will result. To begin, we will seed 50 index cases uniformly throughout the year. A straightforward extension is to consider non-uniform seeding to enhance the heterogeneity, but we don't consider this here.

We will represent the effect of the environment by a single, time-varying field. For simplicity, we will refer to this field as the temperature, T, and we will suppose the temperature is given by

$$T(t) = \cos\left[\frac{2\pi t}{365}\right]. \tag{D.10}$$

The simulation process is as follows

1. We seed a pre-specified number of outbreaks uniformly throughout the year.
2. For each index case we generate a reproduction number $R_e(t)$, based on when the case occurs, and introduce R_e new cases at a time $t_{i+1} = t_i + 20$.
3. We recursively introduce new cases for each infection until the transmission chain ends or all new cases occur after day 365.
4. We calculate the sample weights for each case using the procedure described in §D.3.

The function $R_e(t)$ will determine the degree of temporal heterogeneity in reproduction number. We specify an $R_e(t)$ that is continuous in time. When a non-integer reproduction number is predicted, we sample for a discrete R_e using a draw from a Bernoulli distribution; that is,

$$R_e = \mathrm{floor(R_e) + X}, \qquad \text{where} \qquad \mathrm{X \sim Bernoulli(R_e - floor(R_e))}, \tag{D.11}$$

and floor is a function that rounds a continuous number down to the nearest integer.

The result of this simulation process is a sample of line-list data, where each case has a time, a sample weight, and a R_e value. We will then perform a linear regression to try and estimate the parameters of the data generating process. We will assume a model of the form

$$R_e = a + bT(t) + \epsilon, \qquad \text{and,} \qquad \epsilon \sim \mathcal{N}(0, 0.3), \tag{D.12}$$

where ϵ is observation noise. We will fit for the intercept, a, and slope, b.

To test our ability to cover the true model, we will consider two different true models. The first is when the true model is of the exact same form as the assumed model, (D.12). In this case, the true model is given by

$$R_e = \bar{a} + \bar{b}T(t), \tag{D.13}$$

where $\bar{a} = 1.3$ and $\bar{b} = 1$ are constant. Ideally, after we fit model (D.12), we find that $a = \bar{a}$ and $b = \bar{b}$

The second case is when the assumed model is mis-specified. In this case the true model is slightly more complicated. We will assume that the slope for temperature is not actually constant and that the true model is given by

$$R_e = \hat{a} + \hat{b}(t)T(t) + \epsilon \qquad \text{and,} \qquad \mathrm{\hat{b}(t) = \hat{b}_0 + \hat{b}_1 \cos\left[\frac{2\pi t}{365}\right]}, \tag{D.14}$$

where $\hat{a} = 1.3$, $\hat{b}_0 = 1$, and $\hat{b}_1 = 0.5$ are constants. In this case, we hope to find that $a = \hat{a}$. Additionally, the best we can hope for (from the perspective of model accuracy for random, unseen cases) is that $b = \mathbb{E}\left[\hat{b}(t)\right]$, where $\mathbb{E}\left[\hat{b}(t)\right]$ is the average value of $\hat{b}(t)$ over time, and is given by

$$\mathbb{E}\left[\hat{b}(t)\right] = \frac{1}{365}\int_0^{365} \hat{b}(t)\mathrm{d}t. \tag{D.15}$$

Because we have specified an even variation over the time domain, we simply find that $\mathbb{E}\left[\hat{b}(t)\right] = \hat{b}_0$. Thus, we hope that over an ensemble of simulations, $b \to \hat{b}_0$.

D.4.1 Key findings

The two experiments outlined in §D.4 elucidate the bias–variance trade off between the unweighted and weighted methods.

If the model is exactly the same as the data generating process, then we should not use weights in the regression because there is no source of bias and the variance of the weighted method is higher. In this idealised case all observations provide perfect information about the underlying model. Weighting suppresses the information provided by some points relative to others. In this example all points are perfectly informative, so down-weighting effectively reduces the amount of information we extract from the data. This is detrimental to the model in a similar manner to having fewer observations; that is, we are not maximising the information extracted from the data. A consequence of this is that when we perform a regression using observations generated with the exact form of the model we are fitting to, the variance (uncertainty) of the weighted predictions is higher than that of the unweighted predictions. This is illustrated in Figure D.1. Figure D.1a shows the distribution of predicted errors (differences between predicted and true parameters) when data is generated using the model defined by Equation (D.13). For both the intercept and slope predictions, the unweighted model performs better; the variance in prediction is smaller and the prediction is unbiased. Figure D.1b shows how the mean error in the predicted slope changes with total number of iterations. In this case, the only real difference between methods is the variance in the estimator.

If the data generating process is different from the assumed model (which is more realistic scenario) not adjusting for sampling bias will lead to biased estimates. This is illustrated in Figure D.2. Figure D.2a illustrates the distribution of predicted errors when data is generated by the model defined by Equation (D.15). The distribution of errors for the predicted slope using the unweighted model is not centered at 0; that is, the estimate is biased. In contrast, the distribution of errors for the predicted slope using the weighted regression model is centered at 0. The contrast is even more stark in Figure D.2b. The bias in the estimate for the unweighted regression is evident as the mean error does not converge to zero, unlike that of the weighted regression. In this case the mean slope is 1, so the bias of around -0.1 for the unweighted regression is a relative error of around 10%[7].

It is worth emphasising that the residuals will be correlated, and the fundamental source of bias is the different abundance of correlated residuals. This correlation may be introduced by some underlying, unobserved random effects (which is often the assumption when performing geospatial regression with a spatial random field). But a fundamental component of the introduced bias is a result of preferential sampling, in part due to the data generating process itself. So proposing a model with a spatio-temporally correlated random field would partially mitigate the bias by allowing for spatially correlated residuals; however, it will not eliminate the bias because it will not address the over abundance of some clusters of correlated residuals relative to others.

Since we have no way of knowing how much our assumed model deviates from the *true* model, we must *pick our poison*. Using a weighted regression will increase the variance in our prediction, but reduce the bias: bias or variance. If we have confidence that our proposed model is very similar to the true model, we would not accept this increase in variance. Otherwise, we would prefer to use

[7] The unweighted method underestimating the receptivity is not a surprise, lower R_e children will always outnumber their higher R_e parents.

an unbiased estimator. By applying both methods to the real observations, and performing careful[8] out-of-sample validation, we can contrast the outputs and select the most appopriate method.

The estimator we devised using weighted regression was unbiased for this problem; however, there will be differences between this toy problem and the real world data generating process. We attempted to include enough of the dynamics to be representative, but simple enough to illustrate the fundamental principal; that being, preferential sampling induced bias that can be adjusted for using weighting.

D.5 Testing this approach on real data

We can test the effect of likelihood-weighting on the observational data from Vietnam. To do this, we will use the geospatial model detailed in the main text and Appendix E

We will use hold-out sets to evaluate the out-of-sample performance with and without weights. A new question arises: how do you evaluate model performance using inherently biased data? We would expect the unweighted model to perform better overall on random hold-out sets because the hold-out data likely comes from the over-represented classes, which will also be over represented in the training data. Thus, the apparent predictive power of the unweighted method will be high because we are still training the model on the same type of data; however, the lower variance will give the unweighted method an apparent edge over the weighted method. To reliably compare the two approaches using hold-out sets, we must be selective when constructing hold out sets. In this section we will compare a random approach (k-fold cross-validation) and a selective approach in which we use hold-out sets by chosen region. For the selective approach, we hold out data from a single region, train both weighted and unweighted models on the remaining data, and then make predictions for the data in the hold out set. We do this for all regions with at least 5 observations of P.*falciparum* and P.*vivax*. We summarise the out-of-sample prediction accuracy using the root mean square error (RMSE).

The RMSE's for the k-fold cross validation analysis are contained in Table 1. We see that using k-fold cross validation for both parasites the unweighted regression is consistently more accurate at out-of-sample prediction. This result, as discussed, was expected. For both parasites, the out-of-sample prediction accuracy was slightly smaller for every fold using an unweighted method.

The difference in RMSE between the weighted and unweighted methods for region-based hold out sets is illustrated in Figure D.3. The difference between methods is most stark for P.*falciparum*. The unweighted model was more accurate for out-of-sample prediction in the regions surrounding the Krông Pa district; although, surprisingly, not in the Krông Pa district itself (which has the most data of any region). The weighted method was more accurate in all other regions. This heterogeneity illustrates an important point; the weighted method will likely perform comparatively worse than the unweighted method in areas where more of the data has been down-weighted. In this case, Krông Pa and the surrounding districts are areas of high importation rate (as illustrated in Figure 6 in the main text). This will lead to smaller weights on these observations, and a model that performs better for other districts.

Validation using region-based hold out sets illustrates that the weighted method performs better for regions outside of places with a high density of observations. In other words, by weighting the observations we prevent over fitting to preferentially sampled regions, and the train a more generalisable model.

E Geospatial model for R_e

The R_e predictions given by the diffusion network model are continuous estimates of integer quantities. If 10 case are equally likely to be the source of infection of one other case then each potential source will have $R_e = 0.1$; however, in reality one of the cases had $R_e = 1$ and the other 9 had $R_e = 0$. The diffusion network model will predict integer R_e values when there is no confusion about the source–target relationships (*e.g.*, there is only one plausible source or there are no plausible subsequent infections)[9]. This clarity is more likely in places with fewer cases and when there

[8] As outlined in §D.5

[9] or by chance if there is an integer multiple of "next-generation" cases

Fold	P.*falciparum*		P.*vivax*	
	Weighted	Unweighted	Weighted	Unweighted
1	0.83	0.79	0.54	0.54
2	0.73	0.70	0.39	0.39
3	0.72	0.69	0.31	0.31
4	0.83	0.84	0.52	0.52
5	0.73	0.66	0.66	0.66
6	0.86	0.81	0.66	0.65
7	0.87	0.81	0.35	0.35
8	0.86	0.86	0.47	0.47
9	0.81	0.81	0.39	0.39
10	0.69	0.64	0.35	0.34

Table 1: The results for k-fold cross validation for each parasite comparing the weighted and unweighted methods. The entries are the root-mean-squared errors (RMSE) for the test data set for each fold. The unweighted method performs better under k-fold cross validation.

is no "next-generation" such that $R_e = 0$. The later of these scenarios in particular is the most common source of integer predictions. We can see in Figure E.1 that the count for data that have $R_e = 0$ is markedly different from any other value (including other integer values).

We can view this data as the result of a superposition of two data generating processes: the first being making integer predictions when the transmission chain is clear and the second making continuous predictions if there is any confusion. Given the abundance of $R_e = 0$ in the histogram of Figure E.1, and how unremarkable any of the other integer values are, we can simplify this dichotomy into (i) a process that results in $R_e = 0$ and (ii) a process that results in $R_e > 0$. Such processes are modeled by *zero-inflated* models.

A zero-inflated model comprises two parts: a *zero model* predicting the likelihood of a zero occurring and a *continuous model* that predicts the value of R_e conditional on $R \neq 0$. We use a zero-inflated Gamma (ZIG) model. The probability that $R_e = 0$ is given by

$$\mathbb{P}(R_e = 0) = \pi, \tag{E.1}$$

and for $R_e > 0$, the conditional probability is given by

$$\mathbb{P}(R_e = x | R_e > 0) = (1 - \pi) Gamma(x; \alpha, \beta), \tag{E.2}$$

where α and β are the shape and scale parameters of the Gamma distribution respectively.

The zero component is modelled using a Bernoulli process. If we introduce a binary variable Z such $Z = 1$ if $R_e = 0$ and $Z = 0$ otherwise, we have

$$Z \sim Bernoulli(\pi). \tag{E.3}$$

We use logistic regression to estimate π such that

$$logit(\pi) = \theta_0 + \mathbf{X}^T \boldsymbol{\theta}_0, \tag{E.4}$$

where θ is the intercept of the zero model, $\mathbf{X}$ is a vector of predictors, and $\boldsymbol{\theta}_0$ is a vector of coefficients of the zero model.

The same set of features, $\mathbf{X}$, informs the continuous model (E.2). We use a logarithmic link functions such that the mean of the *Gamma* function is given by

$$\log\left(\frac{\alpha}{\beta}\right) = \theta + \mathbf{X}_i^T \boldsymbol{\theta}, \tag{E.5}$$

where θ is the intercept and $\boldsymbol{\theta}$ is the vector of coefficients for the continuous model.

We consider a range of static and dynamic covariates—covariates at a monthly resolution—that are thought to influence transmission intensity. These include rainfall (CHIRPS), vegetation (EVI), Tasselled cap brightness (TCB), Tasselled cap wetness (TCW), whether or not there is

forest at that point (FOREST), elevation, accessibility to cities, temperature suitability indices for P.*falciparum* or P.*vivax* (TSI_Pf or TSI_Pv respectively), land surface temperature observations during the day (LST_day), the night (LST_night), and the mean diurnal difference (LST_delta). For an alternative geospatial formulation see [4]. For each dynamic covariate, we extract the value observed at the month of symptom onset. We also extract one month either side of the observation; denoting the month before by *lag* and the month after by *lead*. Typically, models will not include the *lead* covariates, possibly because such a model cannot be used for predicting in the future (where the lead values are unknown). In a model for receptivity, however, the reproduction number of a case that is infectious now will depend on the suitability for transmission during the subsequent infectious window. In contrast, other metrics such as prevalence is a consequence of the historic conditions for transmission.

We use a rudimentary wrapper-based variable selection method to whittle down the full covariate set and prevent over-fitting. We start with all covariates including lagged/leading variables and the appropriate temperature suitability index for the parasite being modelled. We first filter out covariates that have little correlation with the observation, dropping covariates that have correlations with R_e less than 0.05 in magnitude. We then fit a model with the remaining N covariates and calculate the Aikake Information Criteria (AIC). We then fit N models, each model corresponding to the full model with a different covariate removed, and calculate the AIC for each model. We consider the reduced model with the lowest AIC. If this AIC is lower than that of the full model, we replace the full set of covariates with the reduced set corresponding to this model; that is, we drop the covariate that provides the greatest drop in AIC. We iterativelly repeat this analysis, at each iteration dropping the covariate that results in the biggest decrease in AIC until no further improvement is attained by dropping a single covariate. We use the subsequent reduced set of covariates as the final model. This optimisation procedure is a so-called *greedy* method, using locally optimal steps to find a locally optimal value. There is no guarantee that the final set of covariates corresponds to the global minimum AIC over all possible choices of covariate. The final reduced set of covariates for each parasite can be found in Table 2 in the main text.

It is advantageous to weight each observation during the regression as argued in §D. We use a vector of weights $\mathbf{w} = (w_1, ..., w_n)$, a weight for each observation, to modify the total log-likelihood function. We truncate the values of $\mathbf{w}$ between the 5% and 95% quantiles to mitigate the influence of outlier weights. The log-likelihood for a single observation, assuming the model defined by (§E.1–§E.5), is given by

$$\ell_i((R_e)_i|\pi, \alpha, \beta) = \begin{cases} \ln(\pi), & \text{if } R_e = 0 \\ \ln(1-\pi) + (k-1)\ln((R_e)_i) - (R_e)_i - \ln(\Gamma(k)), & \text{if } R_e > 0. \end{cases} \tag{E.6}$$

So, the weighted total log-likelihood is given by

$$\ell(k, 1) = \sum_i w_i \ell_i((R_e)_i|\pi, \alpha, \beta). \tag{E.7}$$

In order to calculate the weights $\mathbf{w} = (w_1, ..., w_n)$ using the method outlined in §D.3, we must have first fit the network diffusion model of Appendix §B and the importation model of Appendix §C. Thus, there is significant technical overhead required to perform the weighted regression we have outlined here. The advantage, though, is a more robust use of the limited data available.

F Confidence intervals and sensitivity analysis

F.1 Confidence intervals

For each of the geospatial model predictions from Figure 4 in the main text, we illustrate the 95% confidence intervals in Figures F.1, F.2, and F.3.

The risks associated with the upper confidence interval are more severe than those of the lower confidence interval. In the case of each of the three metrics (receptivity, vulnerability, and malariogenic potential), scenarios closer to the upper confidence interval correspond to greater than expected burden. For example, if the realised reproductive number is close to the upper

confidence interval, many parts of Vietnam would experience $R_e > 1$ and, subsequently, growth in case numbers. While this is not the expected behaviour, such scenarios should be factored into risk management when planning for malaria elimination.

F.2 Sensitivity analysis

To test the sensitivity of the predictions, we must consider the entire modelling pipeline. We generally do not make strong assumptions about the parameter values, and let the model fit determine the appropriate value. To evaluate whether the geospatial R_e model is robust to data ommision, see the model validation section in §D, which includes random hold out sets, and regional hold out sets. In this section, we consider a range of prior mean β values (which we will refer to as $\bar{\beta}$) in the network diffusion model, and propagate these predictions through the pipeline to assess any sensitivity to variation.

$\bar{\beta}$ determines the lengthscale overwhich cases may be connected by transmission. A larger $\bar{\beta}$ value means smaller plausible distances of interaction, and a small $\bar{\beta}$ value allows for longer interactions. We consider the values $\bar{\beta} \in \{0.01, 0.001, 0.0005, 0.0001\}$. The value $\bar{\beta} = 0.001$ corresponds to the "moderate movement" scenario detailed by Routledge *et al*[8], and was the value chosen for the main text. The values an order of magnitude larger $\bar{\beta} = 0.01$ and smaller $\bar{\beta} = 0.0001$ correspond to human movement very unlikely and long range movement likely scenarios respectively.

The distribution of R_e changes with $\bar{\beta}$, but not drastically (see figure F.4). For smaller $\bar{\beta}$, the range over which transmission can occur gets larger. So for the smallest value tested, $\bar{\beta} = 0.0001$, we see that there are fewer $R_e = 0$ cases, with a shift to small, but non-zero reproduction number. This is because these cases that were previously too far apart to be the source of infection for subsequent cases, are now plausible, but unlikely sources of infection. In contrast, as $\bar{\beta}$ gets larger (*e.g.*, $\bar{\beta} = 0.01$) the range of interaction is smaller, so a greater proportion of cases are predicted to not be linked to subsequent infections.

To evaluate the impact of this modified distribution of R_e predictions on downstream geospatial models, we must push the predictions for each $\bar{\beta}$ value through the prediction pipeline. When we do this for P.falciparum, we produce the results illustrated in Figure F.5.

We note that while there are some changes to R_e through the middle, and in the north of the country, the quantitative and qualitative differences are small. The key conclusions from the main text stay the same; namely, annually averaged $R_e < 1$, the locations of relatively higher R_e, and the risk factors for higher R_e. Because the regions of differing R_e across the $\bar{\beta}$ values does not correspond with the more vulnerable regions, the subsequent variation in the malariogenic potential illustrated in Figure F.5 is small.

Thus we can conclude that differences in R_e, introduced by varying our prior assumptions about the transmission dynamics (mediated through $\bar{\beta}$) do not lead to substantially different receptivity maps and malariogenic potential maps.

F.2.1 What if all cases are local?

An important distinction for the diffusion network model is whether or not cases are imported. It is possible that some infections that are originally designated *imported* were in fact acquired locally. Because of the spatio-temporal locality assumptions of the diffusion network model (cases can only be the source of infection to other nearby cases) and causality constraints in time (cases can only be the sources of infection for later cases), the impact of correcting for this potential mis-designation would be that local transmission must account for an additional case at a particular place and time. The net effect would be an additional unit of R_e distributed among all potential sources of each mis-specified case.

To test the potential impact of mis-specifying local cased as imported we consider the most extreme case, where all cases are indigenous. When the diffusion network model is fit under this assumption the R_e predictions shift to higher values, which is illustrated in figure F.6. The changes illustrated in this figure, while significant, are relatively minor compared to the extreme level of mis-specification assumed in the data; that is, 1673 P.*falciparum* cases and 702 P.*vivax* cases are incorrectly designated. The mean change in R_e predictions are 0.35 for P.*falciparum* and 0.28 for P.*vivax*. The errors that may be introduced by incorrect designation wll be localised around the

incorrect data, bounded by a total increment of one for each case, and do not compound throughout the model. The model will be most sensitive to incorrect designation in places where most cases have labeled as imported, which is illustrated in Figure 4 of the main text.

Glossary

AIC Aikake Information Criteria. 17

LGCP log-Gaussian Cox model. 5

References

[1] P. Birello, M. Re Fiorentin, B. Wang, V. Colizza, and E. Valdano. Estimates of the reproduction ratio from epidemic surveillance may be biased in spatially structured populations. *Nat. Phys.*, 2024.

[2] M. Gomez-Rodriguez, D. Balduzzi, and B. Schölkopf. Uncovering the temporal dynamics of diffusion networks. In *Proceedings of the 28th International Conference on International Conference on Machine Learning*, ICML'11, page 561–568, Madison, WI, USA, 2011. Omnipress.

[3] M. Gomez-Rodriguez, J. Leskovec, and A. Krause. Inferring networks of diffusion and influence. *ACM Trans. Knowl. Discov. Data*, 5(4), feb 2012.

[4] B. B. Merga, M. B. Moisa, and D. O. Gemeda. Spatial analysis of malaria risk using geospatial techniques in wabi shebele river sub-basin, southeastern ethiopia. *Sustainable Environment*, 10(1):2321681, 2024.

[5] Jr Reiner, R. C., A. Le Menach, S. Kunene, N. Ntshalintshali, M. S. Hsiang, T. A. Perkins, B. Greenhouse, A. J. Tatem, J. M. Cohen, and D. L. Smith. Mapping residual transmission for malaria elimination. *eLife*, 4:e09520, dec 2015.

[6] I. Routledge, J. E. R. Chevéz, Z. M. Cucunubá, M. G. Rodriguez, C. Guinovart, K. B. Gustafson, K. Schneider, P. G. T. Walker, A. C. Ghani, and S. Bhatt. Estimating spatiotemporally varying malaria reproduction numbers in a near elimination setting. *Nature Communications*, 9(1):2476, Jun 2018.

[7] I. Routledge, S. Lai, K. E. Battle, A. C. Ghani, M. Gomez-Rodriguez, K. B. Gustafson, S. Mishra, J. Unwin, J. L. Proctor, A. J. Tatem, Z. Li, and S. Bhatt. Tracking progress towards malaria elimination in china: Individual-level estimates of transmission and its spatiotemporal variation using a diffusion network approach. *PLOS Computational Biology*, 16(3):1–20, 03 2020.

[8] I. Routledge, H. J. T. Unwin, and S. Bhatt. Inference of malaria reproduction numbers in three elimination settings by combining temporal data and distance metrics. *Scientific Reports*, 11(1):14495, Jul 2021.

[9] D. Simpson, J. B. Illian, F. Lindgren, S. H. Sørbye, and H. Rue. Going off grid: computationally efficient inference for log-Gaussian Cox processes. *Biometrika*, 103(1):49–70, 02 2016.

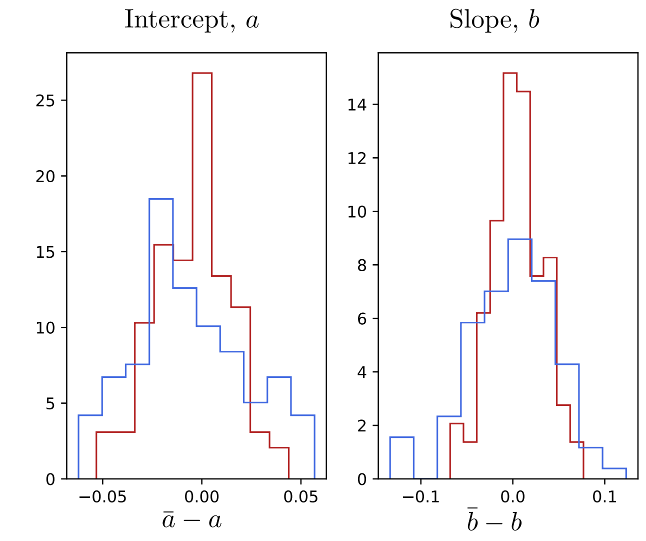

(a)

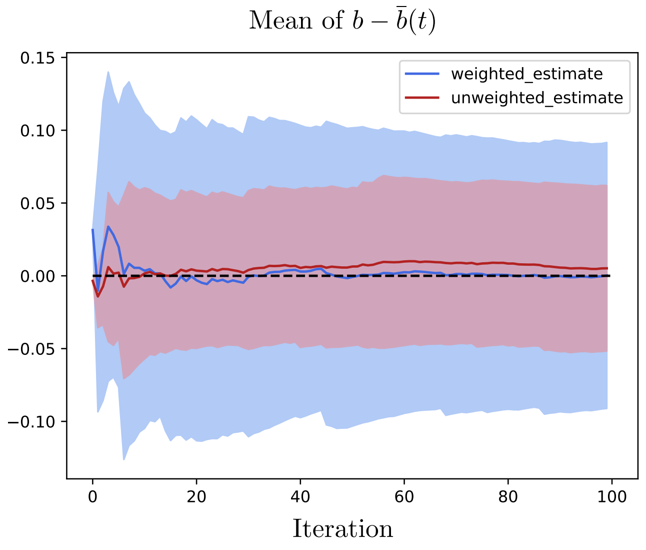

(b)

Figure D.1: (a) Distribution of intercept and slope errors (true minus estimated) for unweighted (blue) and weighted (red) regression When the statistical model is the same as the true model. (b) convergence of error for both regression models as iteration count increases.

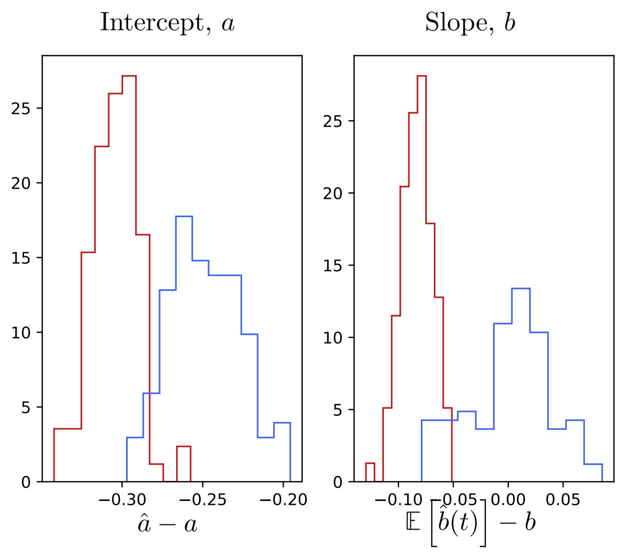

(a)

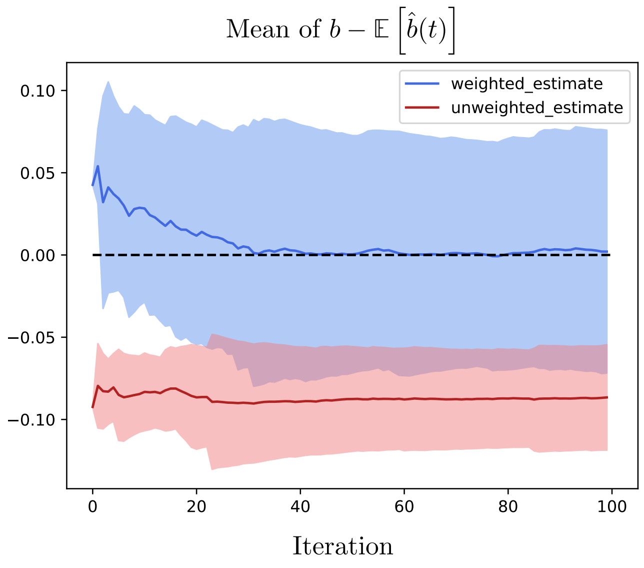

(b)

Figure D.2: (a) Distribution of intercept and slope errors (true minus estimated) for unweighted (red) and weighted (blue) regression when the statistical model is mis-specified with respect to the true model. (b) convergence of error for both regression models as iteration count increases.

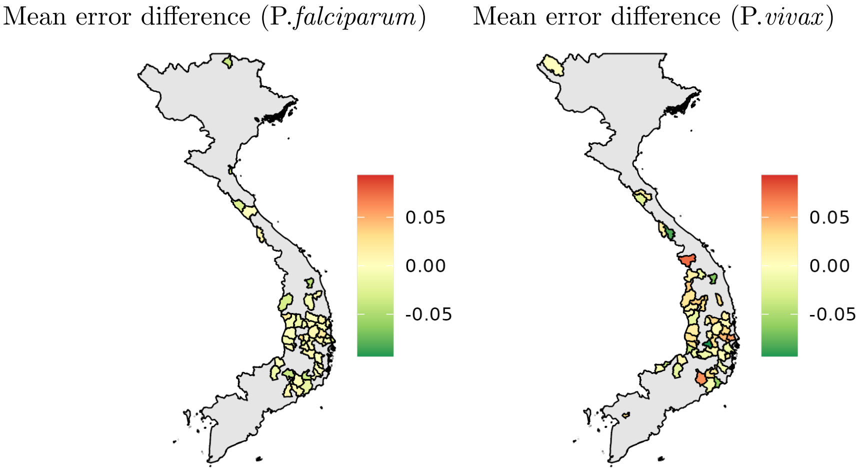

(a) (b)

Figure D.3: Mean difference in prediction error between weighted and unweighted regression methods (weighted - unweighted) for (a) P.*falciparum* and (b) P.*vivax*. A positive difference implies the unweighted method was more accurate and a negative difference implies the weighted method was more accurate.

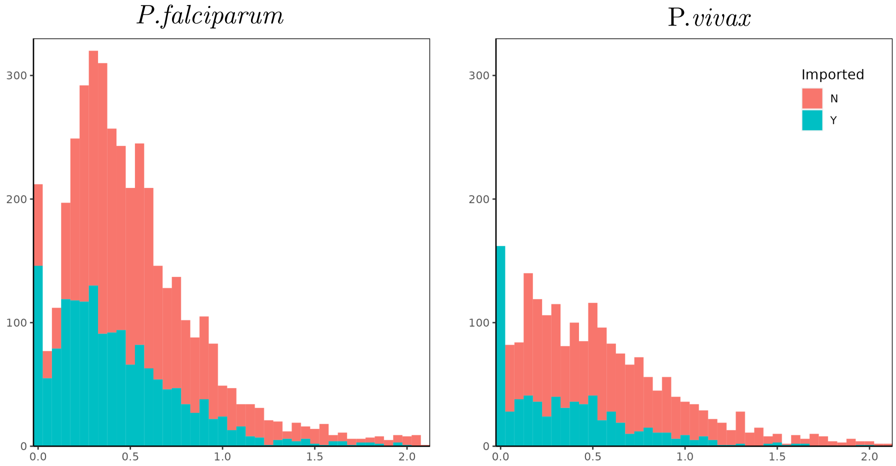

(a) (b)

Figure E.1: Histogram of R_e prediction counts from the diffusion network model for (a) P.*falciparum* and (b) *P.vivax*. There is a clear *jump* at $R_e = 0$, giving the first indication that the process for generating zeroes is different to that of generating $R_e > 0$.

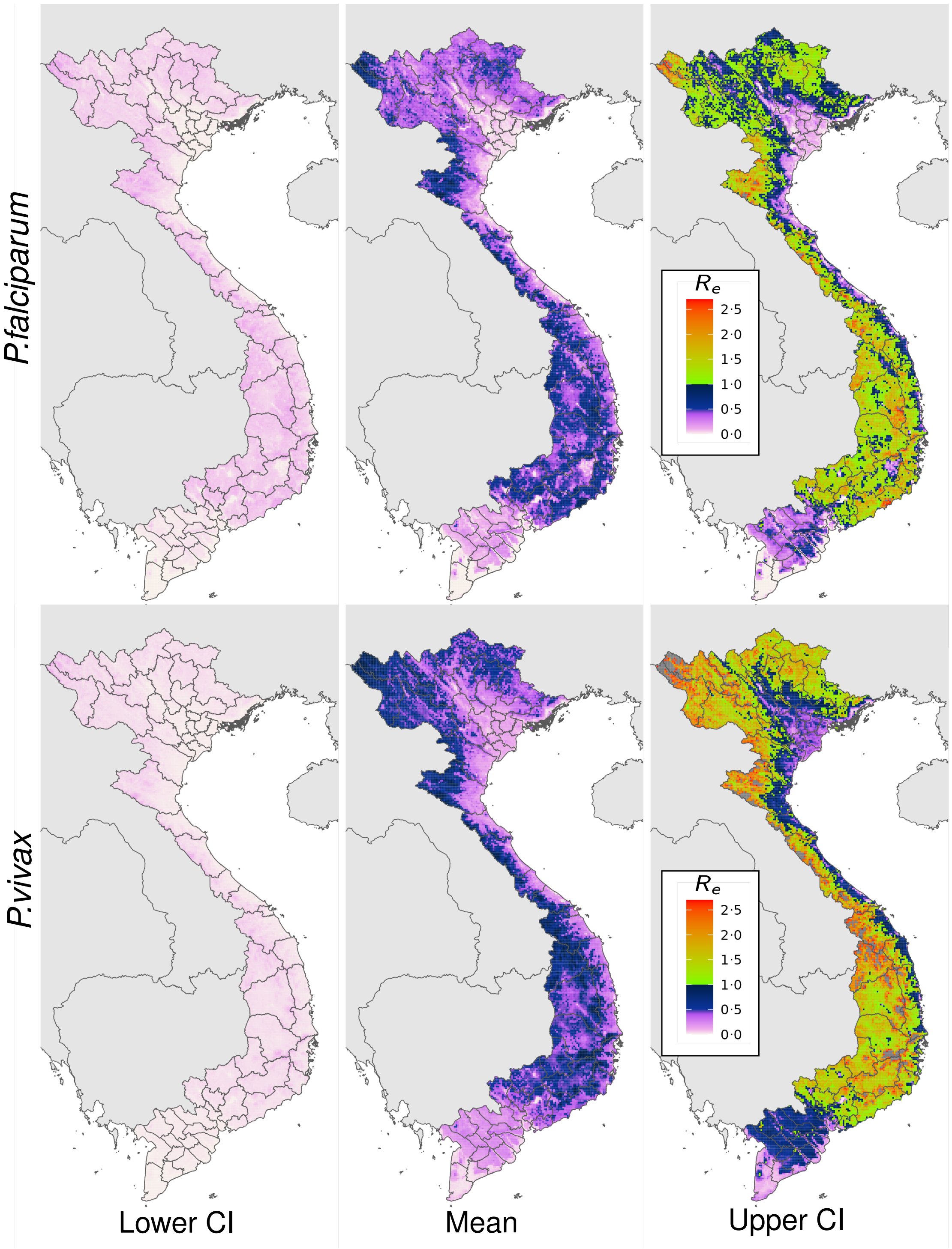

Figure F.1: The 95% confidence intervals for the annual mean of the effective reproduction number, R_e.

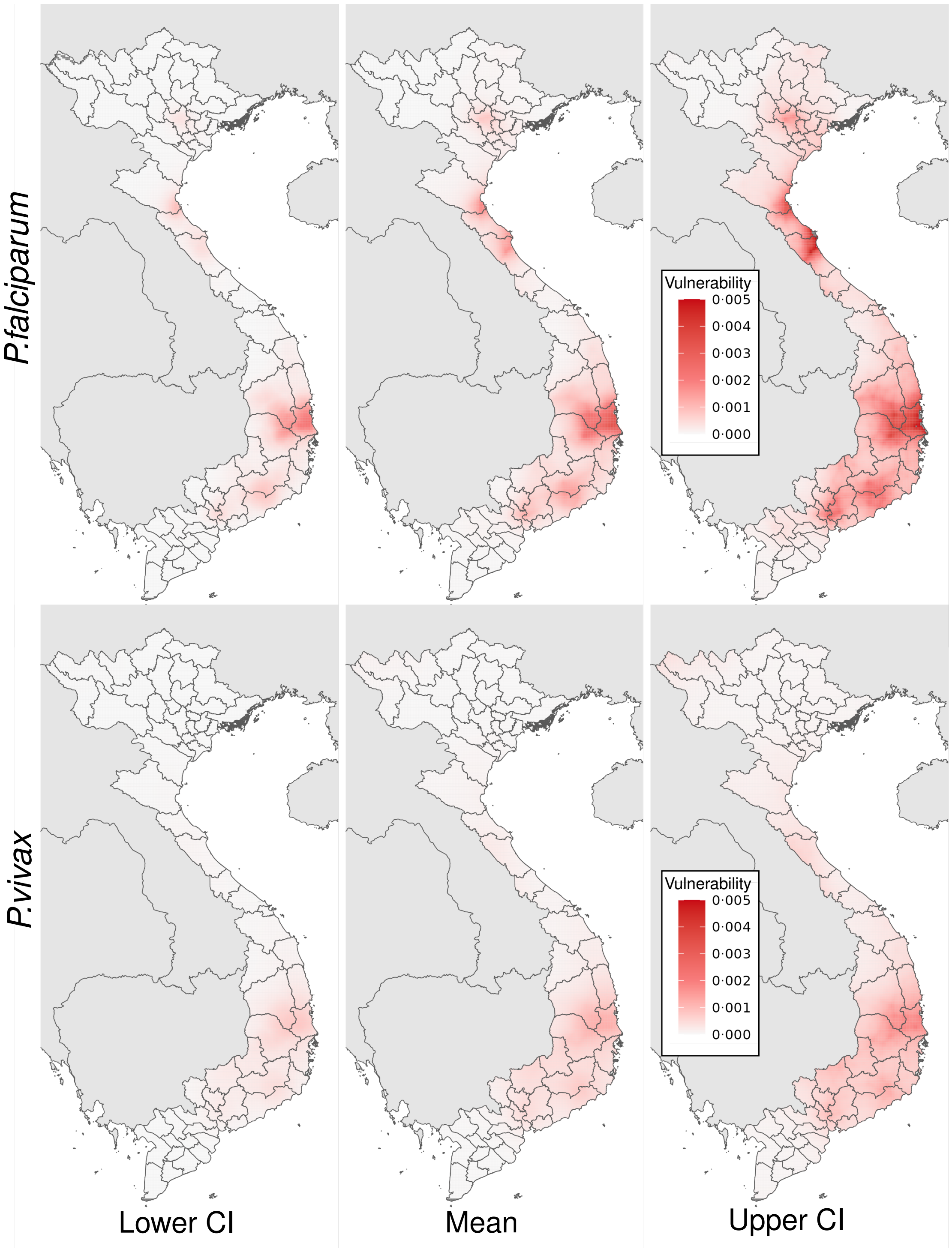

Figure F.2: The 95% confidence intervals for the vulnerability. Note that the range of values in this figure is larger than the main text

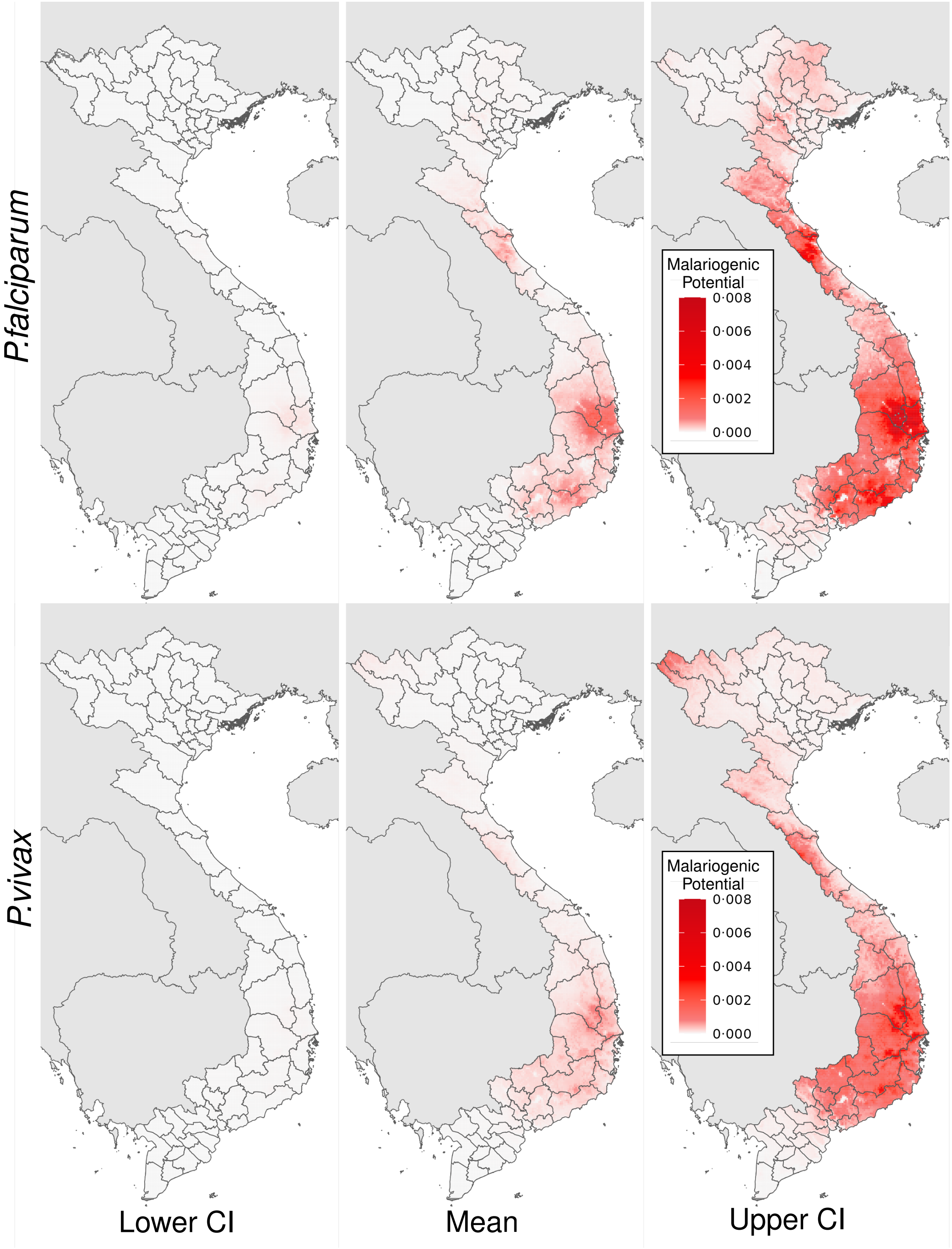

Figure F.3: The 95% confidence intervals for the malariogenic potential

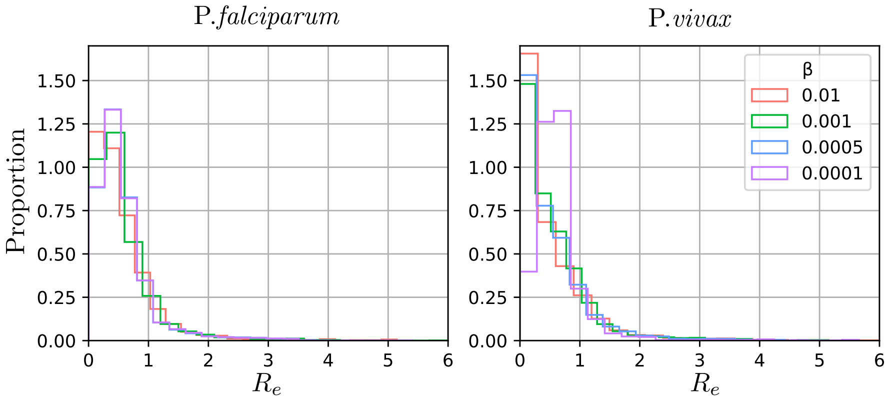

Figure F.4: The distribution of R_e values for $\bar{\beta} \in \{0.01, 0.001, 0.0005, 0.0001\}$ in the network diffusion model for P.*falciparum* (left) and P.*vivax* (right).

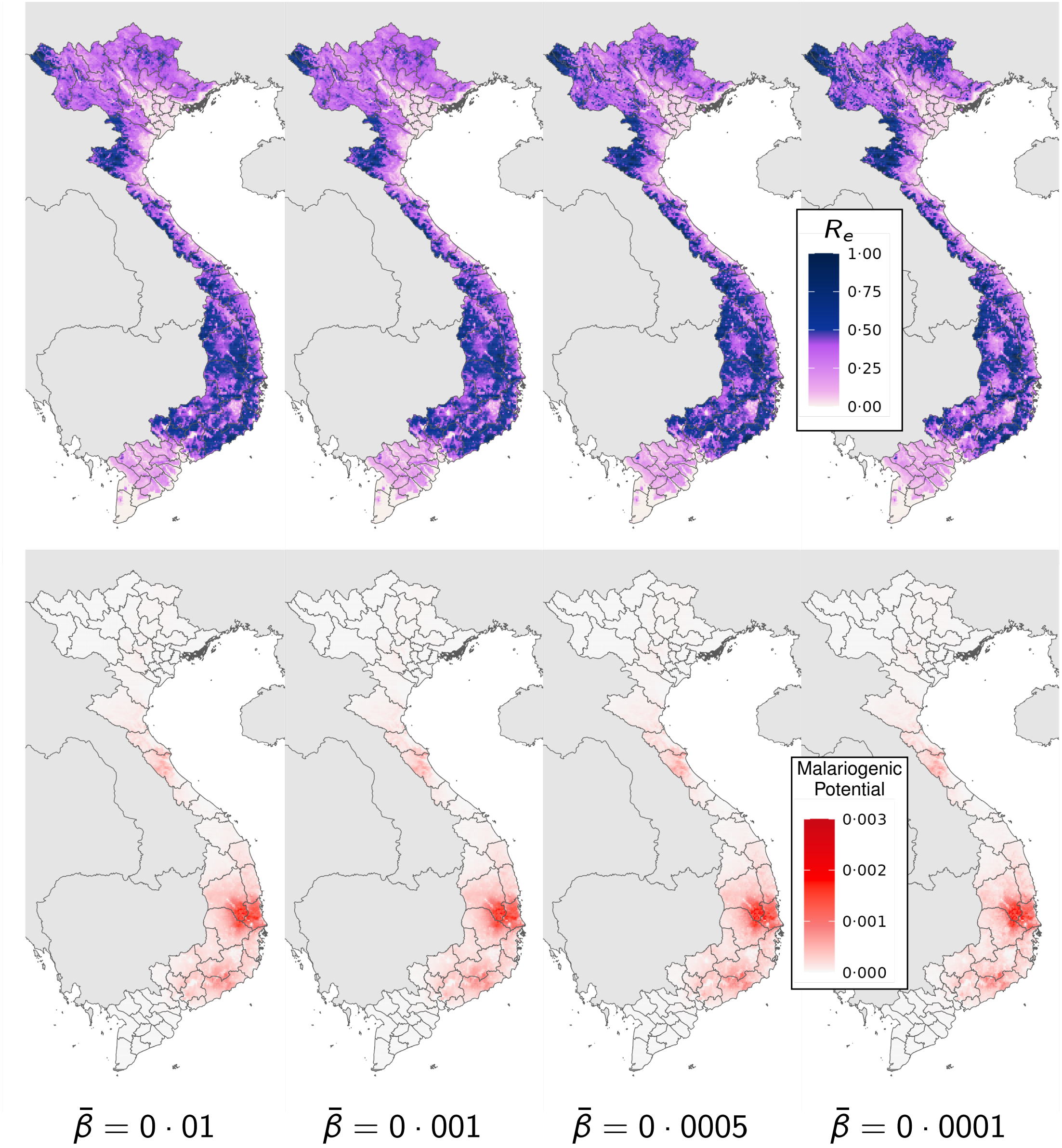

Figure F.5: The receptivity (top row) and malariogenic potential (bottom row) for four different mean $\bar{\beta}$ values in the network diffusion model.

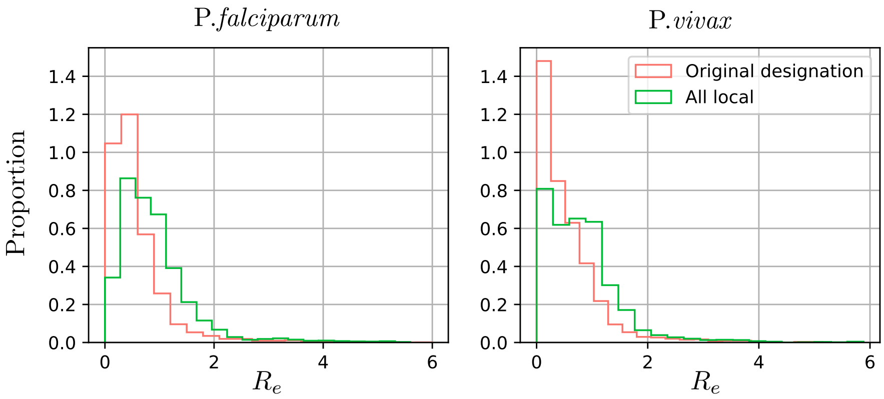

Figure F.6: Distributions of R_e predictions from the diffusion network model based on the original imported/indigenous designation (red) and assuming that all cases are indigenous (green).
